# Supplementary material for: Multicolor hyperafterglow from isolated fluorescence chromophores
Source: Nat Commun. 2023 Jan 30;14:475. doi: 10.1038/s41467-023-36105-y (PMC9884663; doi:10.1038/s41467-023-36105-y)
Supplement: Supplementary file 1 — Supplementary Information [file 41467_2023_36105_MOESM1_ESM.pdf]

Supplementary information

Multicolor hyperafterglow from isolated fluorescence chromophores

Xiao Zhang[1], Mingjian Zeng[1], Yewen Zhang[1], Chenyu Zhang[1], Zhisheng Gao[1], Fei He[1], Xudong Xue[1], Huanhuan Li[1], Ping Li[1], Gaozhan Xie[1], Hui Li[1], Xin Zhang[1], Ningning Guo[1], He Cheng[1], Ansheng Luo[1], Wei Zhao[1], Hui Li[1], Yizhou Zhang[2], Ye Tao [1,*], Runfeng Chen [1,*], Wei Huang[1,3,*]

[1.]State Key Laboratory of Organic Electronics and Information Displays & Institute of Advanced Materials (IAM), Nanjing University of Posts & Telecommunications, Nanjing, 210046, China.

[2.]Institute of Advanced Materials and Flexible Electronics (IAMFE), School of Chemistry and Materials Science, Nanjing University of Information Science and Technology, Nanjing, 210044, China.

[3.]Frontiers Science Center for Flexible Electronics (FSCFE), MIIT Key Laboratory of Flexible Electronics (KLoFE), Northwestern Polytechnical University, Xi'an, 710072, China.

E-mail: iamytao@njupt.edu.cn; iamrfchen@njupt.edu.cn; iamwhuang@njtech.edu.cn.

Content

1. Synthesis and characterization S3

2. Photophysical and morphology investigations S15

3. Hyperafterglow LED and displays S36

1. Synthesis and characterization

Materials: All reagents, unless otherwise specified, were purchased from Energy Chemical, AOB Chem and used without further purification. Manipulations involving air-sensitive reagents were performed in an atmosphere of dry argon (Ar).

Instruments: ^{1}H and ^{13}C-nuclear magnetic resonance (NMR) spectra were recorded on Bruker Ultra Shield Plus 400 MHz instruments with $CDCl_3$ or D_2O as the solvents and tetramethylsilane (TMS) as the internal standard. Aqueous gel permeation chromatography (GPC) was performed on Waters e2695, employing 0.1 mol/L $NaNO_3$ solutions as the mobile phase at the flow rate of 0.8 mL min^{-1}. Powder X-ray diffraction (XRD) patterns were measured using a Bruker D8 Advance diffractometer (Cu Kα: λ=1.5418 Å) under ambient conditions. MALDI-TOF-MS was performed on Bruker autoflex speed MALDI-TOF instrument. Fourier transform infrared spectroscopy (FTIR) was measured using PerkinElmer's Spectrum Two using potassium bromide pressure tablets. Wide-angle X-ray scattering patterns were performed using the Xeuss 2.0 (Xenocs, France) with an incident X-ray Cu-Kα beam (λ = 1.54189 Å). Density functional theory (DFT) and time-dependent DFT (TD-DFT) calculations were performed by using Gaussian 09 D.01 software with B3LYP/6-31g (d) basis set.

Synthesis

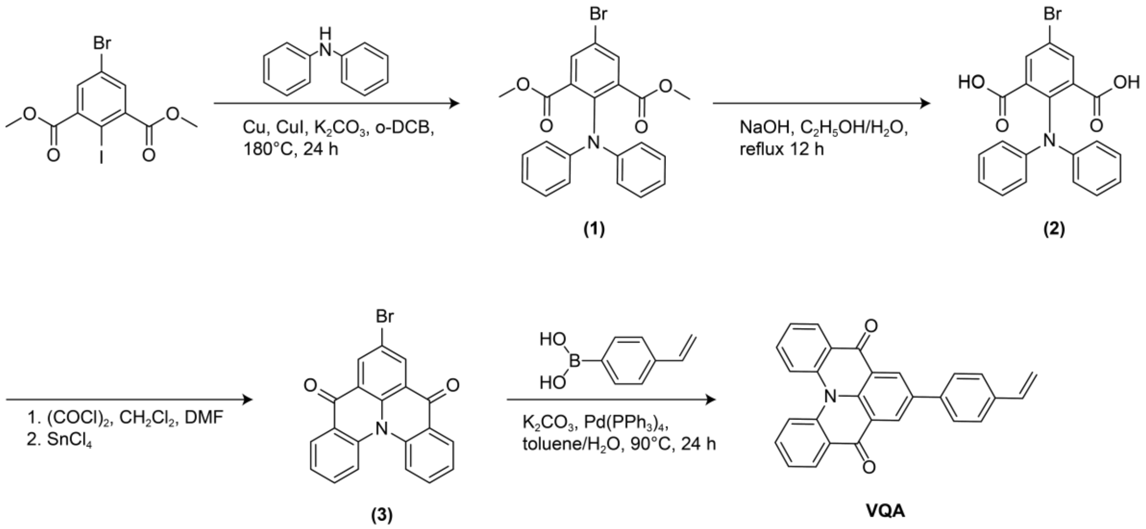

Synthesis of dimethyl 5-bromo-2-(diphenylamino)isophthalate (1)[1]

A mixture of dimethyl 5-bromo-2-iodoisophthalate (3.93 g, 9.85 mmol), diphenylamine (2.00

g, 11.82 mmol), activated copper powder (0.13 g, 1.97 mmol), copper(I)iodide (0.09 g, 0.49 mmol), potassium carbonate (1.77 g, 12.80 mmol) and 30 mL 1, 2-dichlorobenzene (*o*-DCB) was refluxed with stirring for 24 h under an argon atmosphere. After cooling to room temperature, 100 mL of dichloromethane (DCM) was added in the mixture and then stirred 10 minutes. After the mixture was filtered with a glass filter funnel, the filtrate was concentrated under reduced pressure. The given residue was purified through silica gel column chromatography using ethyl acetate (EA)/petroleum ether (PE) (V/V: 1/50) as eluent to give the product as a light green solid product (Yield: 2.81 g, 65%).^{1}H NMR (400 MHz, $CDCl_3$) δ = 7.86 (s, 2H), 7.21–7.17 (m, 4H), 6.97–6.94 (m, 6H), 3.45 (s, 6H). ^{13}C NMR (101 MHz, $CDCl_3$) δ = 166.26, 146.74, 143.57, 136.59, 134.17, 129.03, 122.73, 122.55, 118.01, 52.56. MALDI-TOF: m/z calcd for $C_{22}H_{18}BrNO_4$ $[M]^+$: 440.19; Found: 440.29.

Synthesis of 5-bromo-2-(diphenylamino)isophthalic acid (2)[1]

Dimethyl 5-bromo-2-(diphenylamino)isophthalate (1) (3.50 g, 7.95 mmol) and sodium hydroxide (1.59 g, 39.75 mmol) was added to a solution of ethanol/water (V/V: 1/1, 50 mL). The mixture was refluxed by stirring for 24 h. After cooling to room temperature, acidification with concentrated hydrochloric acid precipitated the yellow solid, which was filtered with a glass filter funnel, washed with deionized water and dried in a vacuum oven overnight. The yellow solid product (Yield: 3.11 g, 95%) was used directly without further purification.

Synthesis of 7-bromoquinolino[3,2,1-*de*]acridine-5,9-dione (3)

Two drops of *N*,*N*-dimethylformamide (DMF) was added to the mixture of 5-bromo-2-(diphenylamino)isophthalic acid (2) (2.60 g, 6.31 mmol) in dry DCM (100 mL) under an argon atmosphere. After adding oxalyl chloride (1.18 mL, 13.88 mmol), the mixture was refluxed for 30 minutes. And then, the Tin(IV) chloride DCM solution (6.94 mL, 2 M, 13.88 mmol) was added and the mixture was refluxed for 3 h. After cooling to room temperature, the mixture was added dropwise to a 1 M aqueous solution of sodium hydroxide and extracted with DCM three times. The organic layer was dried over anhydrous magnesium sulfate. After filtration and solvent evaporation, the given residue was purified through silica gel column chromatography using DCM/PE (V/V: 3/1) as eluent to give the product as an orange-yellow solid product (Yield:

1.71 g, 72%). ^{1}H NMR (400 MHz, $CDCl_3$) δ = 8.81 (s, 2H), 8.48 (dd, J = 7.9, 1.7 Hz, 2H), 8.13 (d, J = 8.6 Hz, 2H), 7.72 (ddd, J = 8.8, 7.2, 1.7 Hz, 2H), 7.53~7.49 (m, 2H). ^{13}C NMR (101 MHz, $CDCl_3$) δ = 117.97, 140.16, 138.41, 135.60, 133.54, 128.47, 126.79, 125.97, 125.49, 120.78, 117.92. MALDI-TOF: m/z calcd for $C_{20}H_{10}BrNO_2$ $[M]^+$: 376.16; Found: 376.20.

Synthesis of 7-(4-vinylphenyl)quinolino[3,2,1-*de*]acridine-5,9-dione (VQA)

To a 50 mL round-bottom flask charged with 7-bromoquinolino[3,2,1-*de*]acridine-5,9-dione (3) (1.00 g, 2.66 mmol), 4-vinylbenzene boric acid (0.59 g, 3.99 mmol), potassium carbonate (0.55 g, 3.99 mmol), toluene (12 mL) and deionized water (2 mL), $Pd(PPh_3)_4$ (0.15 g, 0.13 mmol) by using a syringe under an argon atmosphere. Then, the mixture was heated to 90˚C and refluxed for 24 h. After the mixture was cooled down, 20 mL deionized water was added to the resulting solution and the mixture was extracted with DCM for several times. The organic phase was dried over anhydrous magnesium sulfate. After filtration and solvent evaporation, the given residue was purified through silica gel column chromatography using DCM/PE (V/V: 4/1) as eluent to give the product (VQA) as light yellow solid (Yield: 0.86 g, 81%). ^{1}H NMR (400 MHz, $CDCl_3$) δ = 8.99 (s, 2H), 8.52 (dd, J = 7.9, 1.7 Hz, 2H), 8.16 (d, J = 8.6 Hz, 2H), 7.79 (d, J = 8.3 Hz, 2H), 7.71 (ddd, J = 8.7, 7.0, 1.7 Hz, 2H), 7.56 (d, J = 8.3 Hz, 2H), 7.50 (t, J = 7.3 Hz, 2H), 6.79 (dd, J = 17.6, 10.9 Hz, 1H), 5.87~5.83 (d, 1H), 5.33 (d, J = 11.0 Hz, 1H). ^{13}C NMR (101 MHz, $CDCl_3$) δ = 178.79, 139.86, 138.52, 137.77, 137.62, 136.33, 136.22, 132.93, 130.71, 128.08, 127.35, 127.15, 126.56, 125.41, 124.04, 120.40, 114.72. MALDI-TOF: m/z calcd for $C_{28}H_{17}NO_2$ $[M]^+$: 399.10; Found: 398.96.

Synthesis of 10-(4-vinylphenyl)benzo[9,1]quinolizino[3,4,5,6,7-klmn]phenothiazine-8,12-dione (VQS)

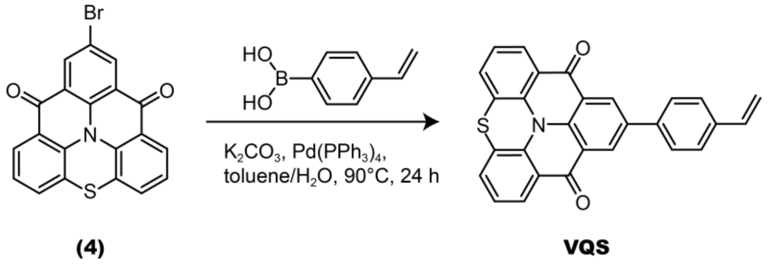

To a 50 mL round-bottom flask charged with 7-bromoquinolino[3,2,1-*de*]acridine-5,9-dione

(4)[2] (1.08 g, 2.66 mmol), 4-vinylbenzene boric acid (0.59 g, 3.99 mmol), potassium carbonate (0.55 g, 3.99 mmol), toluene (12 mL) and deionized water (2 mL), $Pd(PPh_3)_4$ (0.15 g, 0.13 mmol) by using a syringe under an argon atmosphere. Then, the mixture was heated to 95˚C and refluxed for 24 h. After the mixture was cooled down, 20 mL deionized water was added to the resulting solution and the mixture was extracted with DCM for several times. The organic phase was dried over anhydrous magnesium sulfate. After filtration and solvent evaporation, the given residue was purified through silica gel column chromatography using DCM/PE (V/V: 2/1) as eluent to give the product (VQS) as red solid (Yield: 0.76 g, 67%). ^{1}H NMR (400 MHz, $CDCl_3$) δ= 9.20 (s, 2H), 8.26 (dd, J = 6.1, 3.0 Hz, 2H), 7.83 (d, J = 8.0 Hz, 2H), 7.58 (d, J = 8.0 Hz, 2H), 7.31 (m, J = 3.6 Hz, 4H), 6.79 (dd, *J* = 17.7, 10.8 Hz, 1H), 5.86 (d, J = 17.6 Hz, 1H), 5.35 (d, J = 10.9 Hz, 1H). ^{13}C NMR (101 MHz, 10% TFA-d in $CDCl_3$) δ=177.94, 140.96, 138.56, 136.88, 136.36, 136.04, 135.68, 135.29, 133.32, 132.05, 127.30, 126.88, 126.28, 123.52, 122.07, 121.11, 115.34. MALDI-TOF: m/z calcd for $C_{28}H_{15}NO_2S$ [M]+: 429.08; Found: 429.26.

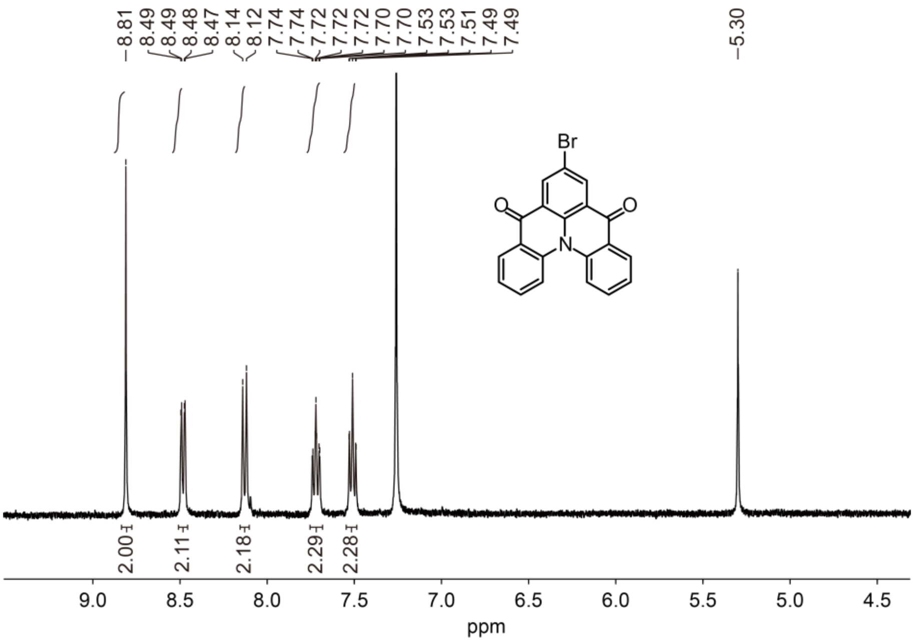

Supplementary Figure 1. ^{1}H NMR spectrum of 7-bromoquinolino[3,2,1-de]acridine-5,9-dione (3) in $CDCl_3$.

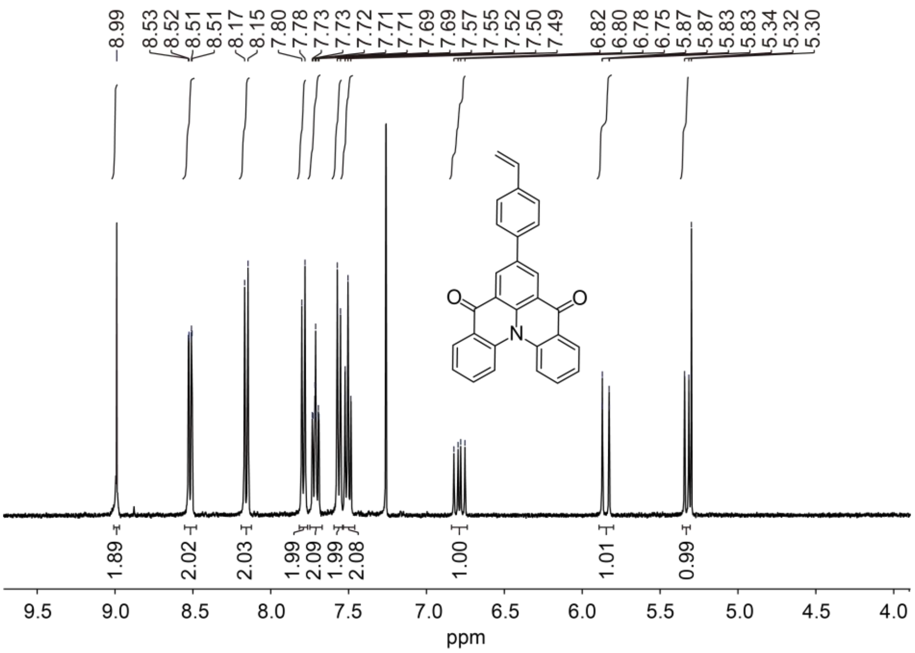

Supplementary Figure 2. ^{1}H NMR spectrum of VQA in $CDCl_3$.

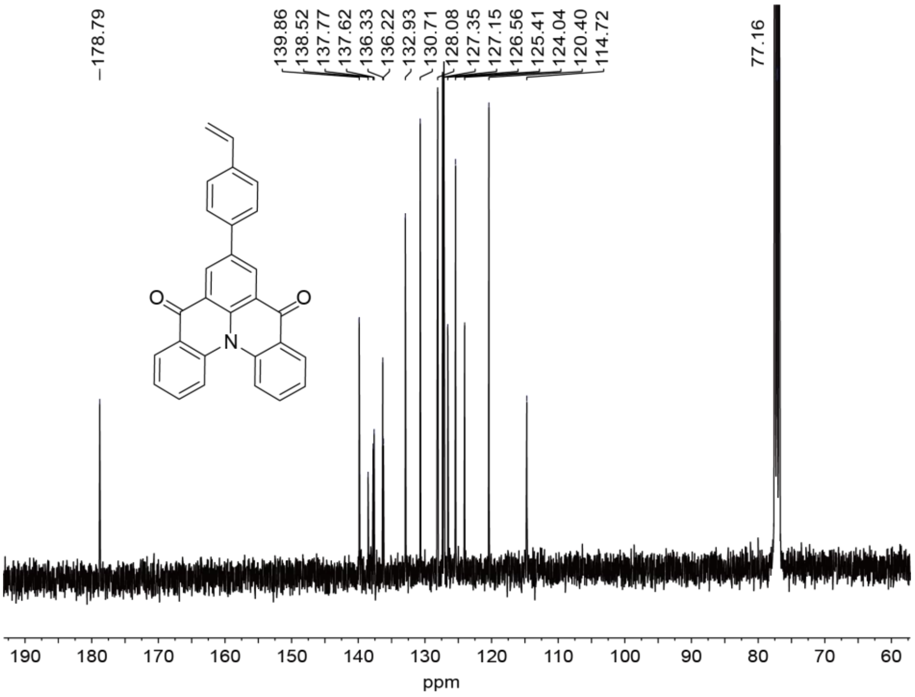

Supplementary Figure 3. ^{13}C NMR spectrum of VQA in $CDCl_3$.

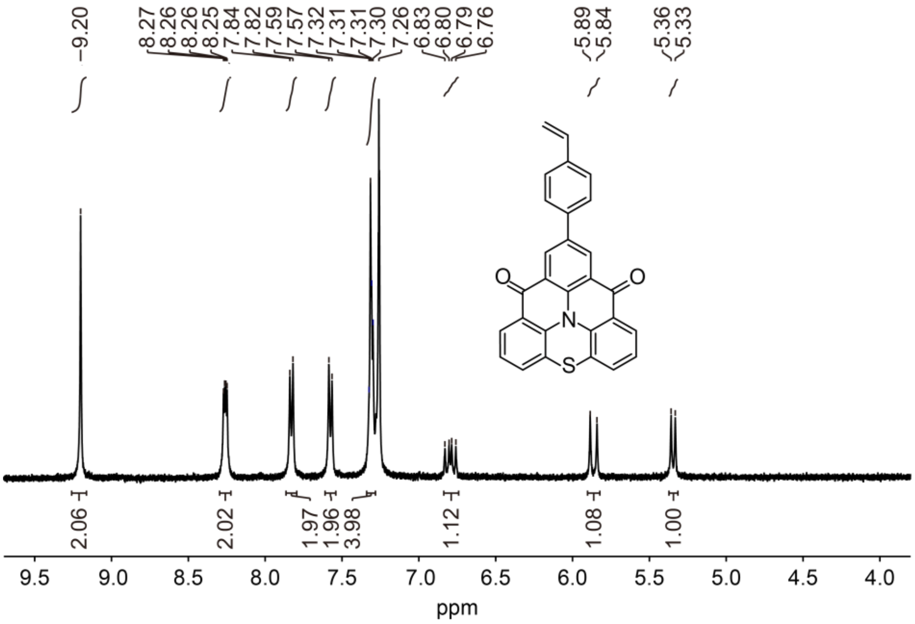

Supplementary Figure 4. ^{1}H NMR spectrum of VQS in $CDCl_3$.

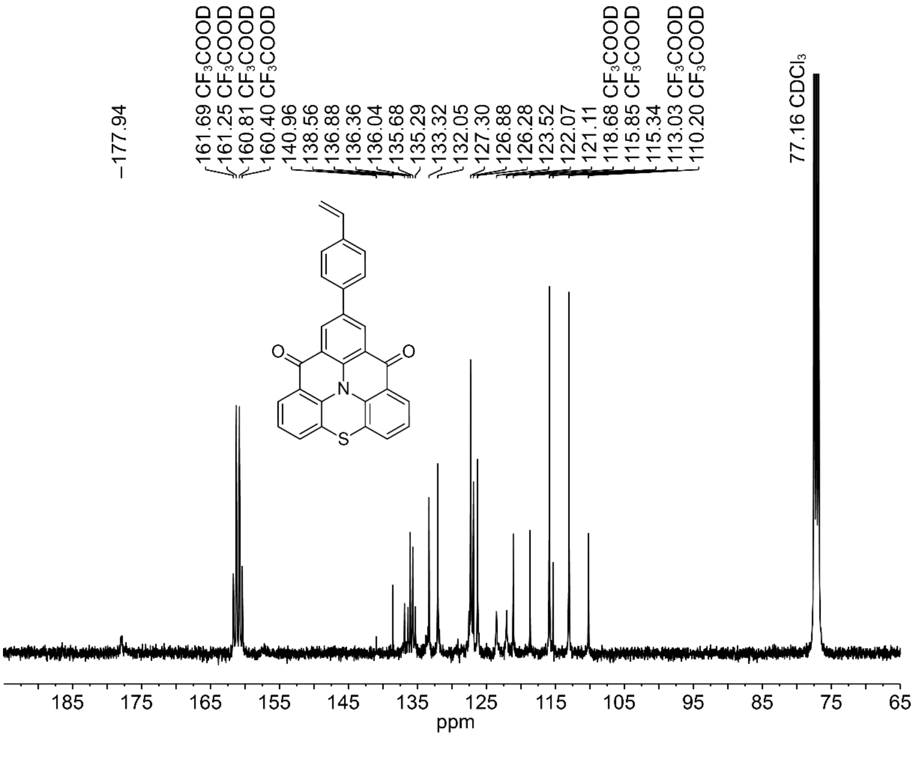

Supplementary Figure 5. ^{13}C spectrum of VQS in 10% TFA-d in $CDCl_3$

General procedure of radical polymerization.

In argon atmosphere, 0.01 equivalent (eq) of 2,2'-azobis(2-methylpropionitrile) (AIBN) and 1.0 eq of vinyl derivative were dissolved in 25 mL freshly distilled tetrahydrofuran (THF). The

mixture was heated to 55°C for 16 h, during which the white, green or red solid was constantly precipitated out from solution. Then, the mixture was cooled to room temperature and added into methanol to precipitate polymeric materials, then the crude product was filtered, followed by washing with PE and DCM, acetone in sequence. Then the solid was dissolved in deionized water and dialyzed by a dialysis tube (molecular weight cut-off = 1000) for 72 h.

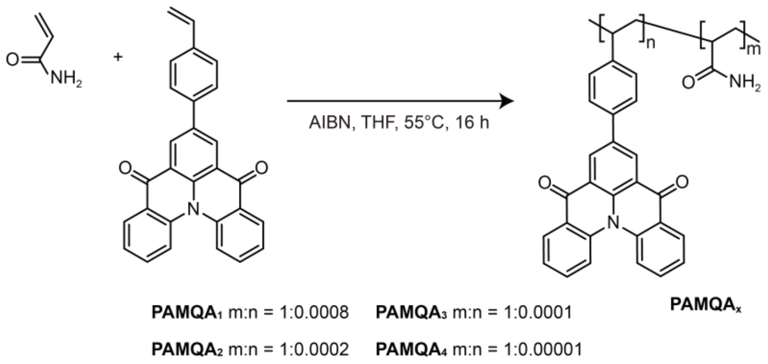

$PAMQA_1$. Following the general procedure of radical polymerization using VQA (15.96 mg, 0.04 mmol, 1.00 eq), acrylamide (3.55 g, 50.0 mmol, 1250 eq), and appropriate amount of AIBN (82.07 mg, 0.5004 mmol, 12.51 eq) in 25 mL freshly distilled THF to afford 3.20 g green powder polymer with a yield of 89.7%. M_n = 7404 Da; M_w = 25512 Da; PDI = 3.44.

$PAMQA_2$. Following the general procedure of radical polymerization using VQA (3.99 mg, 0.01 mmol, 1.00 eq), acrylamide (3.55 g, 50.0 mmol, 5000 eq), and appropriate amount of AIBN (82.02 mg, 0.5001 mmol, 50.51 eq) in 25 mL freshly distilled THF to afford 3.00 g green powder polymer with a yield of 84.4%. M_n = 6651 Da; M_w = 20261 Da; PDI = 3.04.

$PAMQA_3$. Following the general procedure of radical polymerization using VQA (2.00 mg, 0.005 mmol, 1.00 eq), acrylamide (3.55 g, 50.0 mmol, 10000 eq), and appropriate amount of AIBN (82.01mg, 0.50005 mmol, 100.01 eq) in 25 mL freshly distilled THF to afford 3.25 g green powder polymer with a yield of 91.5%. M_n = 7990 Da; M_w = 25268 Da; PDI = 3.16.

$PAMQA_4$. Following the general procedure of radical polymerization using VQA (0.20 mg, 0.0005 mmol, 1.00 eq), acrylamide (3.55 g, 50.0 mmol, 100000 eq), and appropriate amount of AIBN (82.00 mg, 0.500005 mmol, 1000.01 eq) in 25 mL freshly distilled THF to afford 3.10 g

green powder polymer with a yield of 87.3%. M_n = 7937 Da; M_w = 24746 Da; PDI = 3.11.

Polyacrylamide (PAM). Following the general procedure of radical polymerization using acrylamide (3.55 g, 50.0 mmol, 100 eq), and appropriate amount of AIBN (82.00 mg, 0.5 mmol, 1 eq) in 25 mL freshly distilled THF to afford 3.30 g white powder polymer with a yield of 92.9%. M_n = 8973 Da; M_w = 27191 Da; PDI = 3.03.

PAMCzQA. Following the general procedure of radical polymerization using VQA (15.96 mg, 0.04 mmol, 1.00 eq), acrylamide (3.55 g, 50.0 mmol, 1250 eq), vinyl carbazole (96.5 mg, 0.5 mmol, 12.50 eq) and appropriate amount of AIBN (82.90 mg, 0.5054 mmol, 12.635 eq) in 25 mL freshly distilled THF to afford 3.00 g green powder polymer with a yield of 81.9%. M_n = 8284 Da; M_w = 26698 Da; PDI = 3.22.

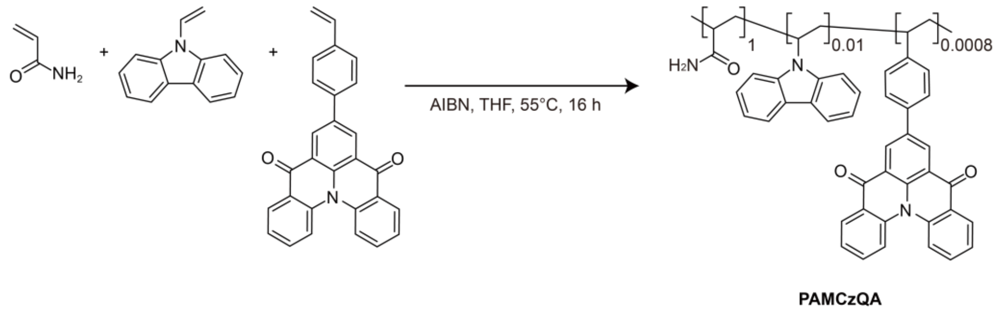

PAMCzQAQS. Following the general procedure of radical polymerization using VQA (15.96 mg, 0.04 mmol, 1.00 eq), VQS (34.33 mg, 0.08 mmol, 2.00 eq), acrylamide (3.55 g, 50.0 mmol, 1250 eq), vinyl carbazole (96.5 mg, 0.5 mmol, 12.50 eq) and appropriate amount of AIBN (83.02 mg, 0.5062 mmol, 12.655 eq) in 25 mL freshly distilled THF to afford 2.8 g red powder polymer with a yield of 75.7%. M_n = 7289 Da; M_w = 15037 Da; PDI = 2.06.

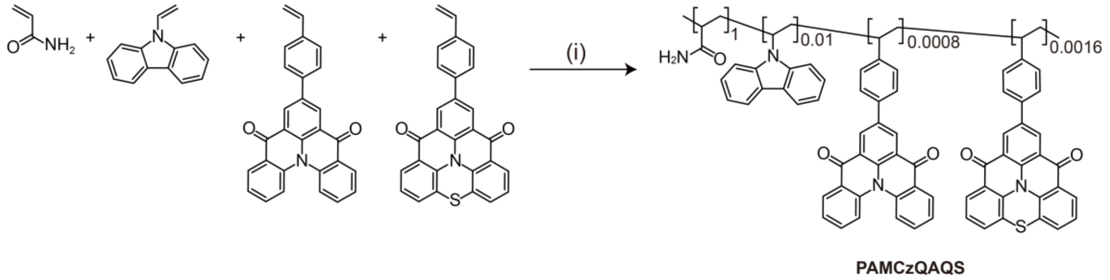

Supplementary Table 1. Characterizations of polymers $PAMQA_x$ (X=1, 2, 3 and 4) by GPC**.**

Polymer	M_n	M_w	PDI
PAM	8973	27191	3.03
$PAMQA_1$	7404	25512	3.44
$PAMQA_2$	6651	20261	3.04
$PAMQA_3$	7990	25268	3.16
$PAMQA_4$	7937	24746	3.11
PAMCzQA	8284	26698	3.22
PAMCzQAQS	7289	15037	2.06

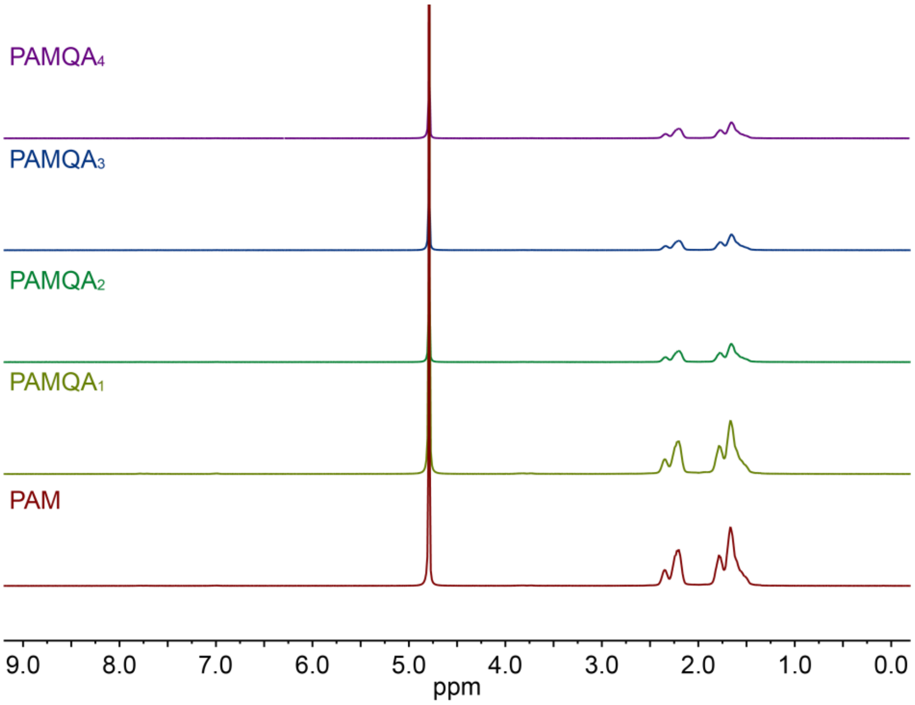

Supplementary Figure 6. ^{1}H NMR spectra of PAM and $PAMQA_x$ (X=1, 2, 3 and 4) in D_2O.

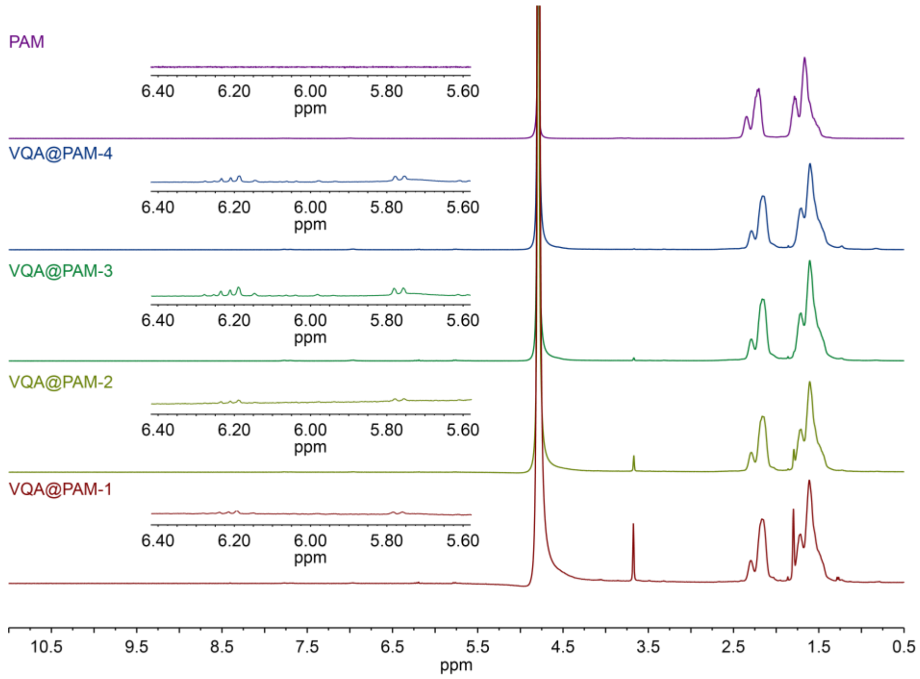

Supplementary Figure 7. ^{1}H spectra of physically blended PAM and VQA in mixed D_2O and d-THF solution with same mass feed ratios to that of $PAMQA_x$.

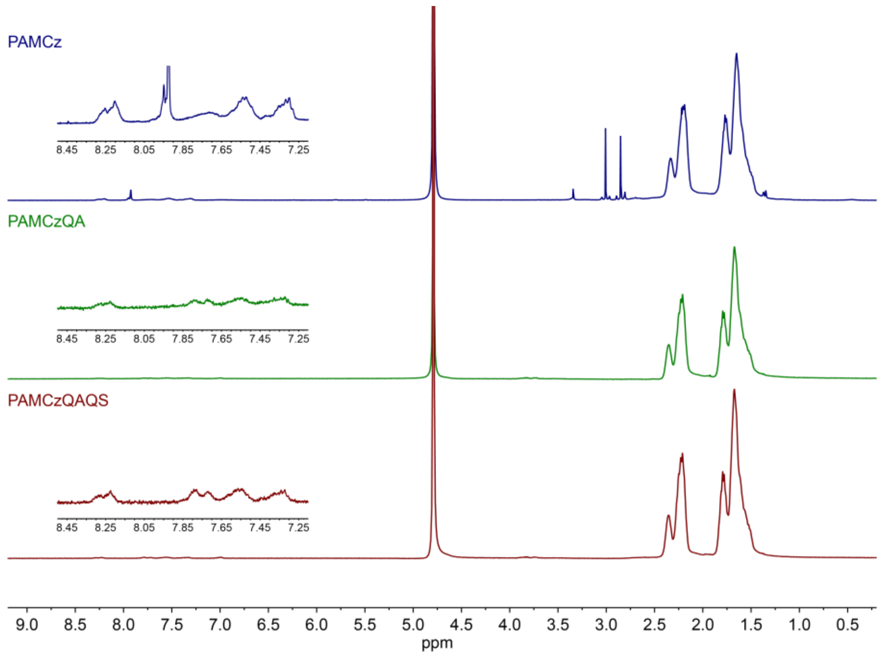

Supplementary Figure 8. ^{1}H NMR spectra of PAMCz, PAMCzQA and PAMCzQAQS in D_2O.

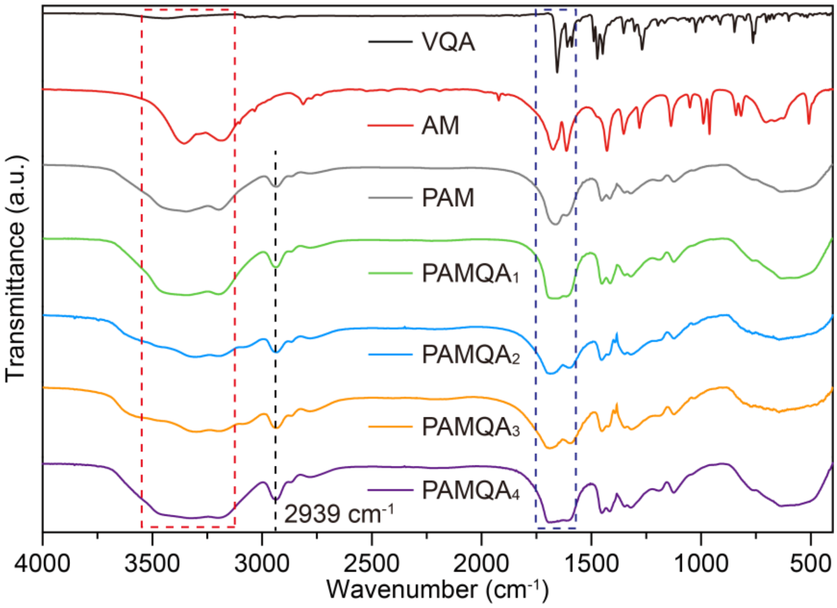

Supplementary Figure 9. FTIR spectra of VQA, AM, PAM and $PAMQA_x$ (X=1, 2, 3 and 4). Compared to AM monomer, the newly emerged peak at 2939 cm^{-1} should be assigned to C-H symmetric and asymmetric stretching vibrations, suggesting the successful polymerization of PAM and $PAMQA_x$; also, the peaks at 3200-3700 cm^{-1} originated from N-H vibration become wider, demonstrating the strong hydrogen-bond interaction between PAM and $PAMQA_x$. Notably, the peaks around 1600 cm^{-1} attributed to the C=O stretching vibrations in $PAMQA_x$ are broader than that in PAM, confirming that the VQA unit is successfully introduced in $PAMQA_x$.

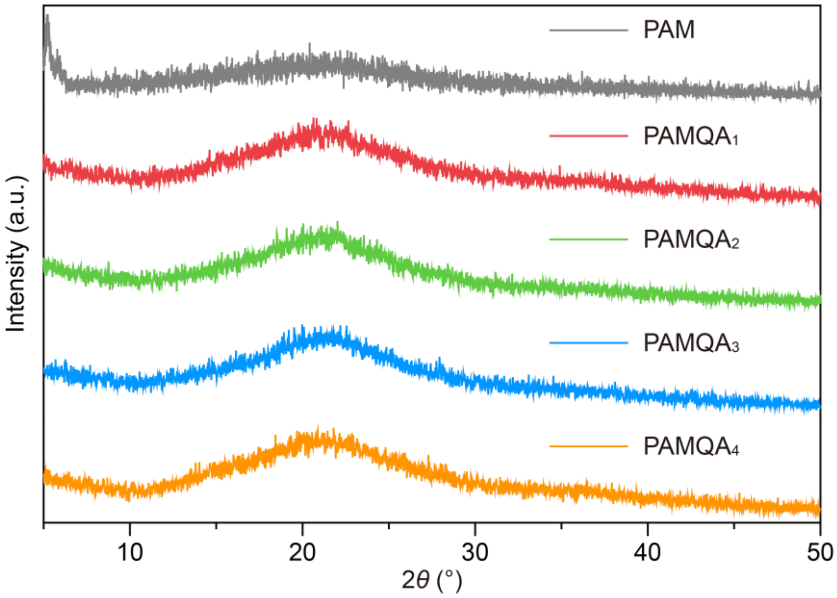

Supplementary Figure 10. Powder XRD spectra of PAM and $PAMQA_x$ (X=1, 2, 3 and 4) films.

General procedures for the preparation of polymer film.

Firstly, 0.6 g $PAMQA_x$ powder was dissolved in 10 mL deionized water followed by the sonication for 10 min under ambient conditions. Subsequently, the mixture was intensely stirred at 60℃ for 1 h to obtain the transparent aqueous solution. Finally, the well-dissolved solution was poured into a clean petri dish and dried at 70℃ in an oven to prepare the amorphous polymer film.

2. Photophysical and morphology investigations

Ultraviolet/visible (UV/Vis) and fluorescence spectra were recorded on a Jasco V-750 spectrophotometer and Edinburgh FLS980, respectively. The absolute photoluminescence quantum yield (PLQY) was obtained using an Edinburgh FLS980 fluorescence spectrophotometer equipped with an integrating sphere. For fluorescence decay measurements, picosecond pulsed light-emitting diode (EPLED-295, wavelength: 300 nm, pulse width: 833.7 ps; VPL-375, wavelength: 375 nm, pulse width: 120 μs) were used. Delayed PL spectra were obtained using an Edinburgh FLS980 fluorescence spectrophotometer with a 10 ms delay time after excitation using a microsecond flash lamp. The microsecond flash lamp produces short, typically a few μs, and high irradiance optical pulses for phosphorescence decay measurements in the range from microseconds to seconds. The delayed PL spectra and ultralong lifetimes were also measured using an Edinburgh FLS980 fluorescence spectrophotometer. Excitation-PL and Excitation-Delayed PL mapping was measured using Hitachi F-4700 with no delay or a 25 ms delay time under ambient conditions.

The lifetimes (τ) of the luminescence were obtained by fitting the decay curve with a multi-exponential decay function of

$$I(t) = \sum_i B_{\mathrm{i}} e^{-\frac{t}{\tau_{\mathrm{i}}}} \quad (1)$$

Where B_i and τ_{i} represent the amplitudes and lifetimes of the individual components for multi-exponential decay profiles, respectively.

The average lifetime was calculated by the function of

$$\tau_{\mathrm{ave}} = \sum_i \varphi_{\mathrm{i}} \tau_{\mathrm{i}} \quad (2)$$

where φ_{i} is the amplitude fraction.

To get the intensity-averaged lifetime (τ_{int}), the $\varphi_{\mathrm{i}}^{\mathrm{int}}$ is defined by the function of

$$\varphi_i^{\mathrm{int}} = \frac{B_{\mathrm{i}} \tau_{\mathrm{i}}}{\sum_i B_{\mathrm{i}} \tau_{\mathrm{i}}} \times 100\% \quad (3)$$

τ_{int} is achieved by the function of

$$\tau_{\mathrm{int}} = \sum_i \varphi_{\mathrm{i}}^{\mathrm{int}} \tau_{\mathrm{i}} \quad (4)$$

To get the amplitude averaged lifetime (τ_{amp}) which was used for the analyses of FRET

process, the φ_i^{amp} is defined by the function of:

$$\varphi_{\mathrm{i}}^{\mathrm{amp}} = \frac{B_{\mathrm{i}}}{\sum_{i} B_{\mathrm{i}}} \times 100\% \quad (5)$$

τ_{amp} is achieved by the function of:

$$\tau_{\mathrm{amp}} = \sum_{i} \varphi_{\mathrm{i}}^{\mathrm{amp}} \tau_{\mathrm{i}} \quad (6)$$ [3]

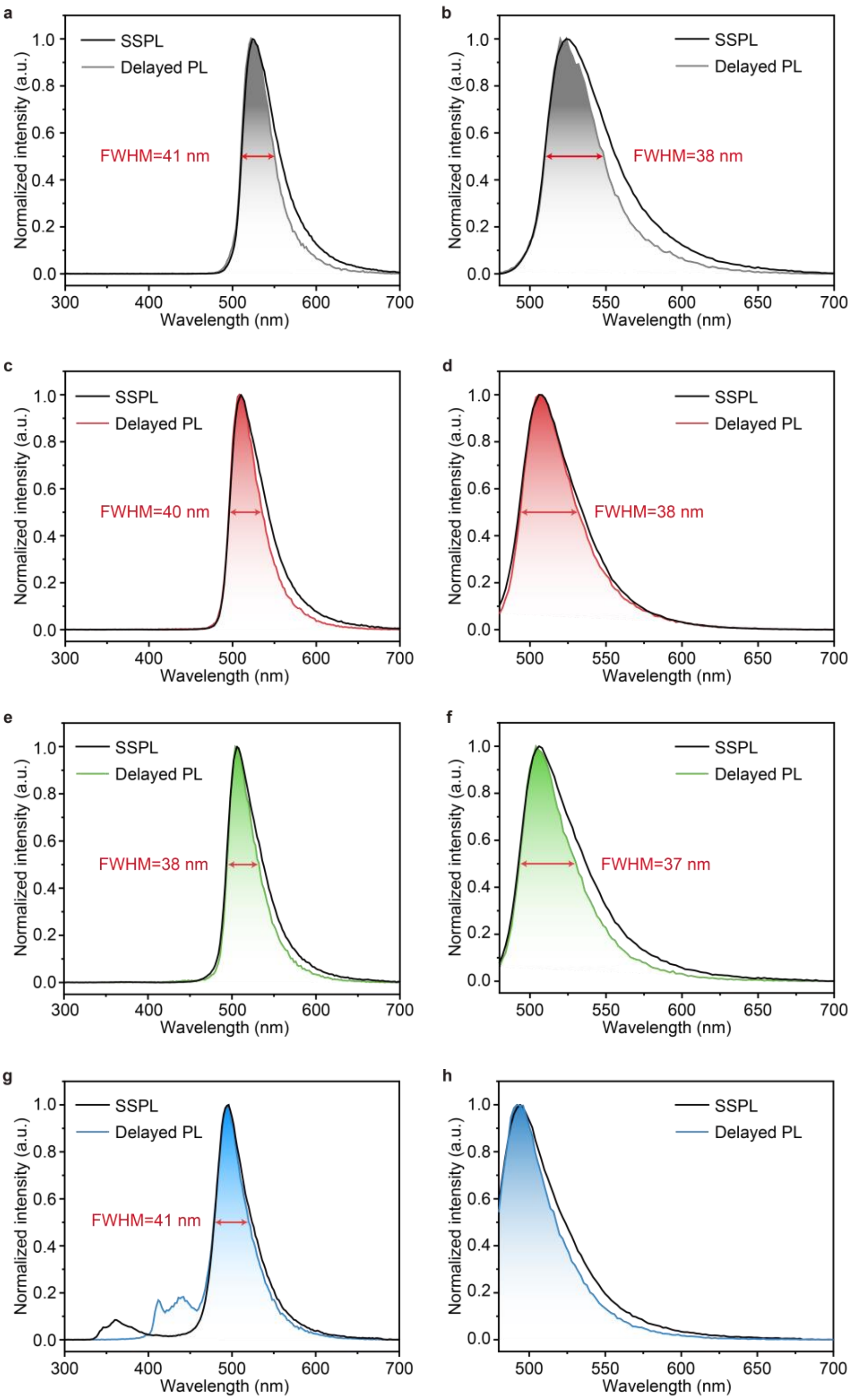

Supplementary Figure 11. SSPL and delayed spectra (10 ms delay) of (a, b) $PAMQA_1$, (c, d) $PAMQA_2$, (e, f) $PAMQA_3$ and (g, h) $PAMQA_4$ films excited by (a, c, e, g) 285 nm UV light and (b, d, f, h) 466 nm visible light under ambient conditions, respectively.

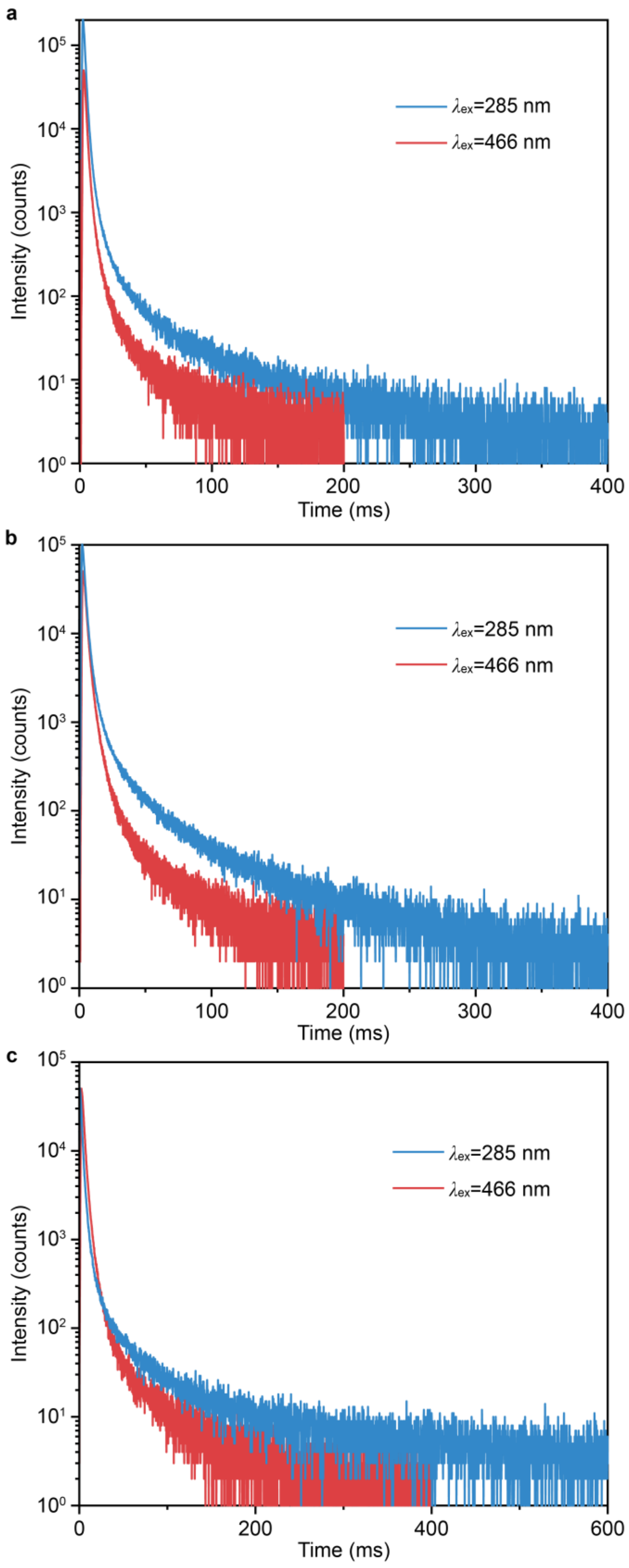

Supplementary Figure 12. Lifetime decay profiles of (a) $PAMQA_1$, (b) $PAMQA_2$ and (c) $PAMQA_4$ films excited by 285 nm UV light and 466 nm visible light under ambient conditions.

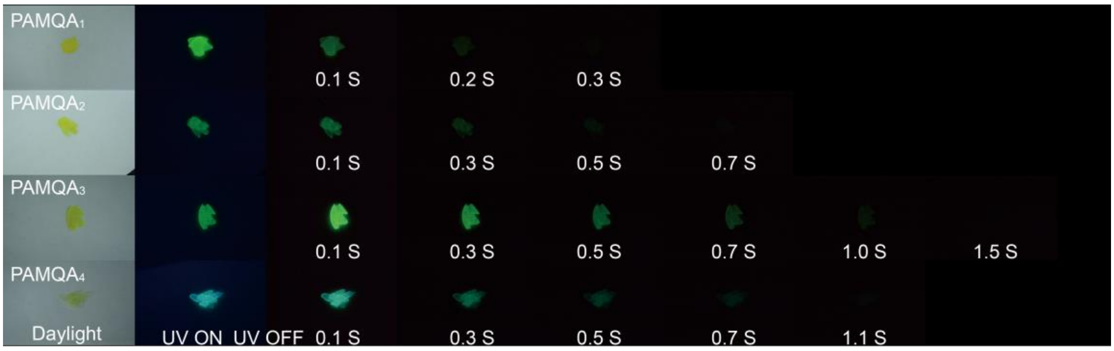

Supplementary Figure 13. Photographs of $PAMQA_x$ (X=1, 2, 3 and 4) films taken after the removal of 365 nm UV light under ambient conditions.

Supplementary Table 2. Photoluminescence properties and lifetime parameters of PAM and hyperafterglow polymers $PAMQA_x$ (X=1, 2, 3 and 4).

Polymer	λ_{ex} [nm]	λ_{em}^{H} [nm]	τ^{H}[ms]	λ_{em}^{G} [nm]	τ^{G}[ms]	FWHM [nm]
PAM	285	438	325.15	/	/	/
$PAMQA_1$	285	438	23.96	524	31.20	41
	466	438	/	524	11.86	38
$PAMQA_2$	285	438	26.73	514	36.13	40
	466	438	/	514	14.57	38
$PAMQA_3$	285	438	38.85	504	49.25	38
	466	438	/	504	15.66	37
$PAMQA_4$	285	438	68.15	496	65.92	41
	466	438	/	496	25.65	/

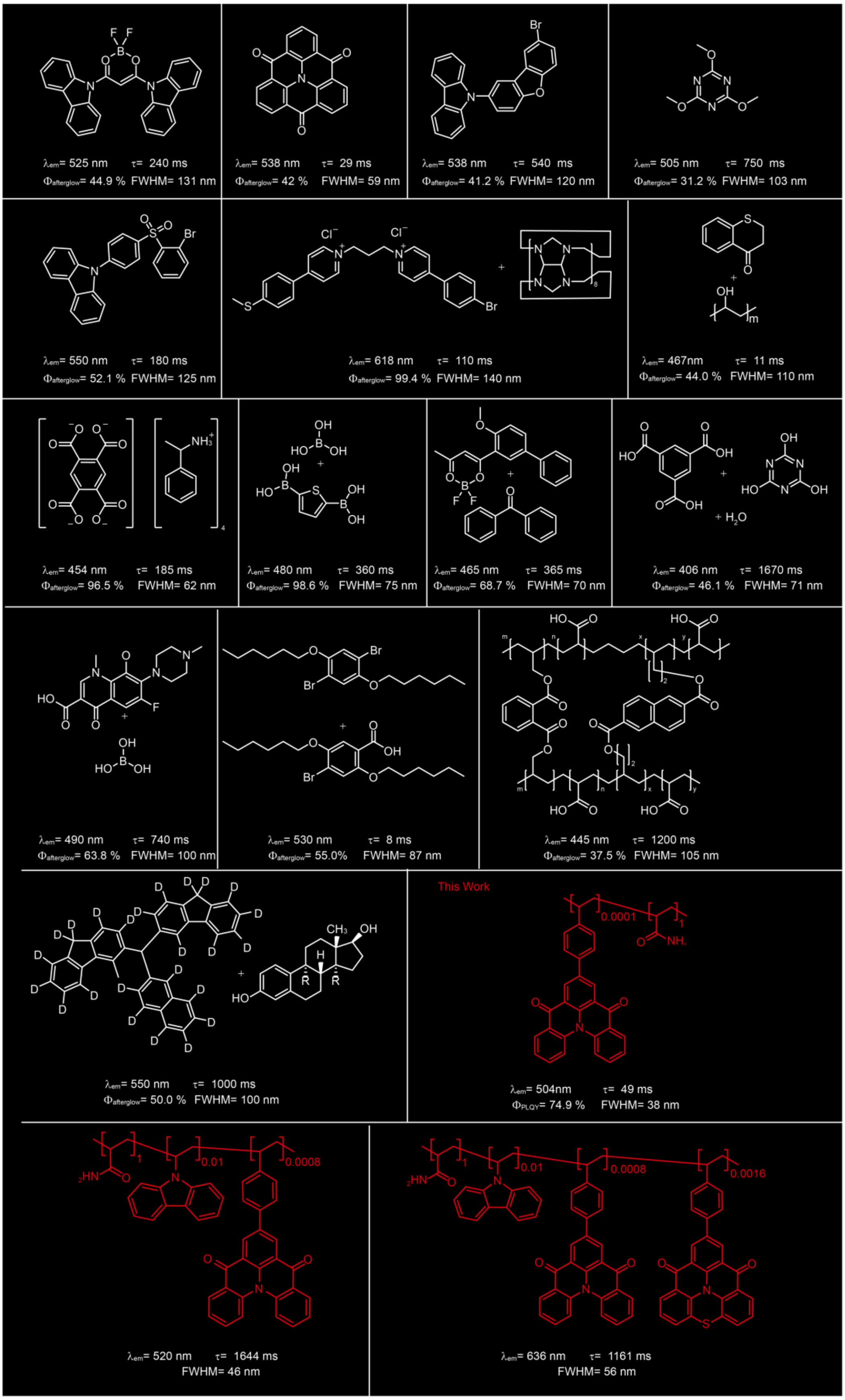

Supplementary Figure 14. The reported high-performance pure organic afterglow materials with corresponding lifetimes, afterglow efficiencies and FWHM under ambient conditions[4-18].

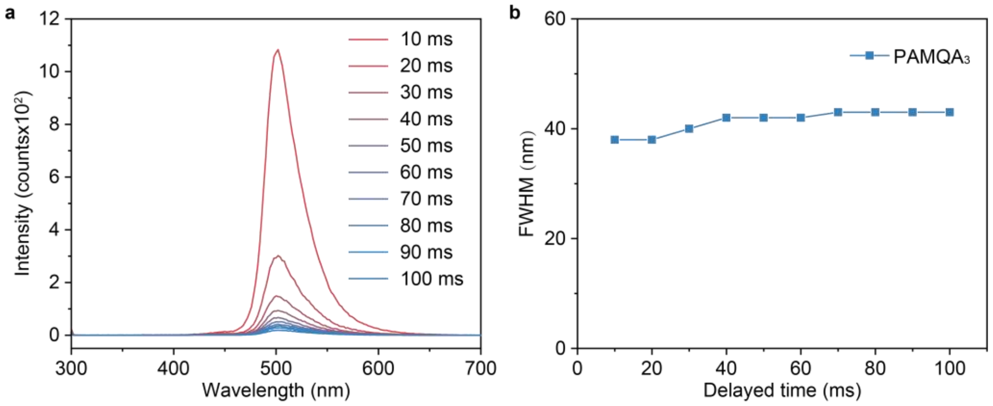

Supplementary Figure 15. (a) Delayed PL spectra of $PAMQA_3$ film with different delayed time; (b)The afterglow FWHM of $PAMQA_3$ film with different delayed time.

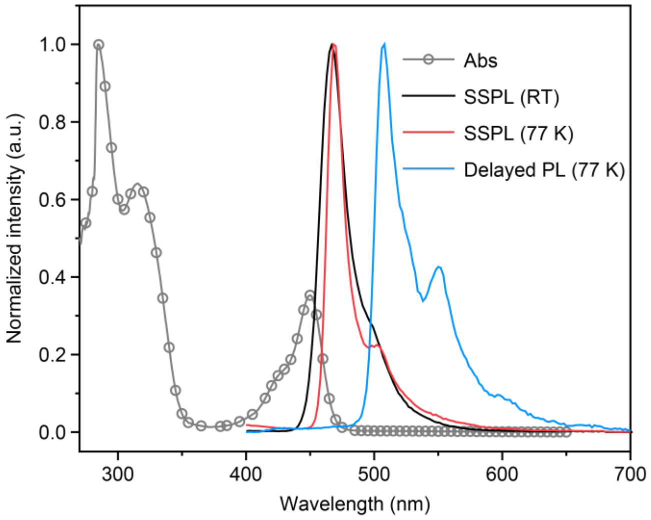

Supplementary Figure 16. UV-visible absorption, steady-state photoluminescence (SSPL) and delayed (10 ms delay) spectra of VQA in toluene (~ 10^{-5} mol L^{-1}) recorded under room temperature (RT) and 77 K. The singlet-triplet energy splitting (ΔE_{ST}) value for VQA was estimated to be 0.20 eV, by the difference between S_1 (468 nm) and T_1 (508 nm) in energy.

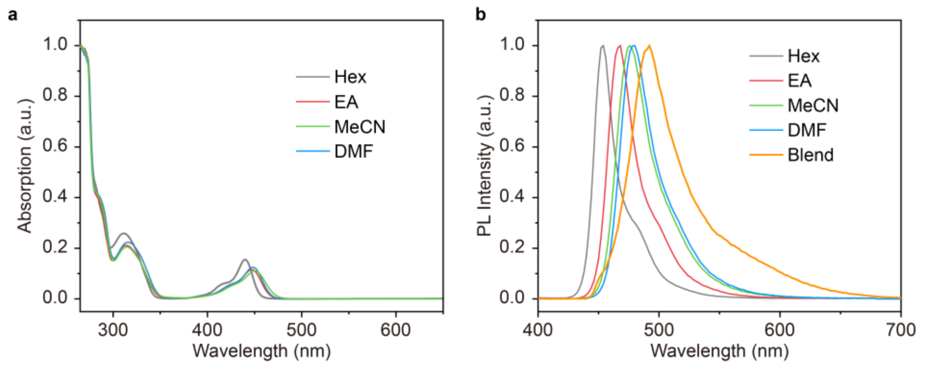

Supplementary Figure 17. Normalized (a) absorption and (b) SSPL spectra of VQA in n-hexane (Hex), ethyl acetate (EA), *N*, *N*-Dimethylformamide (DMF), acetonitrile (MeCN) and PAM film.

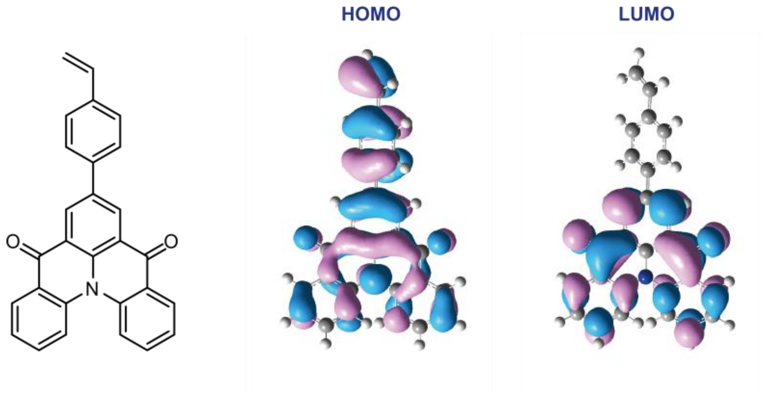

Supplementary Figure 18. Chemical structure, calculated distributions of frontier molecular orbitals (FMOs) distributions of VQA, (isovalue=0.02).

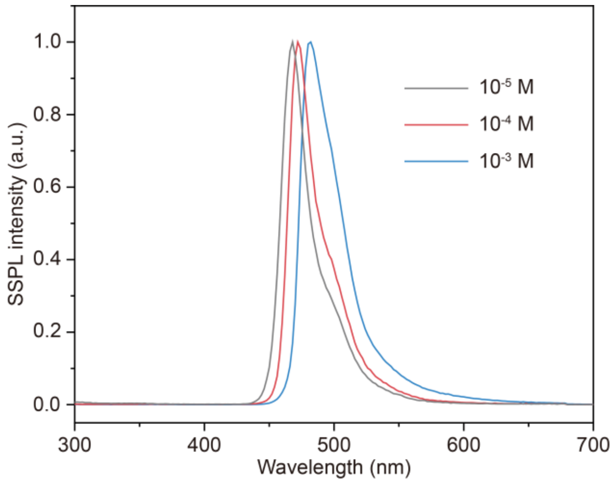

Supplementary Figure 19. SSPL spectra of VQA in toluene solution with different concentrations excited by 285 nm UV light under ambient conditions.

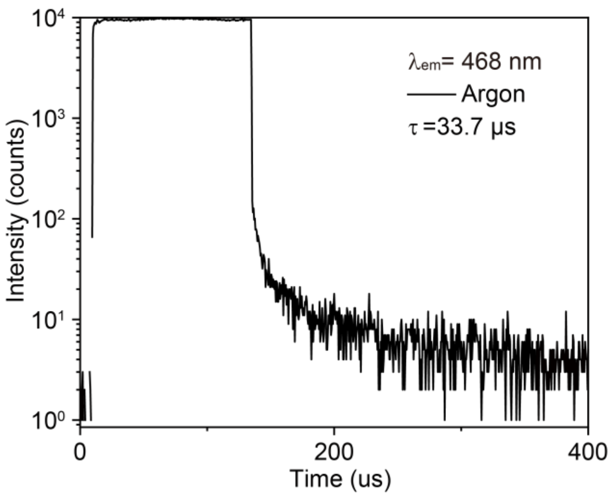

Supplementary Figure 20. Lifetime decay profiles of VQA in toluene (~ 10^{-5} mol L^{-1}) excited by 375 nm UV light under argon conditions.

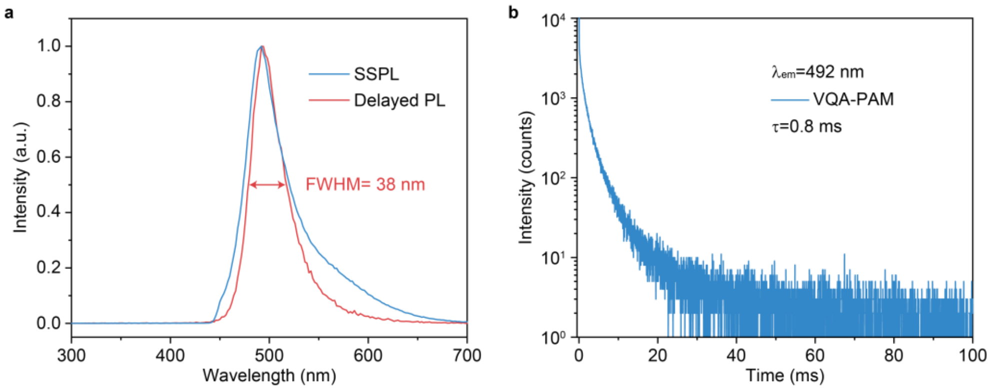

Supplementary Figure 21. (a) SSPL and delayed PL spectra (10 ms delay) and (b) lifetime decay profiles of VQA-PAM (1‰ wt VQA in PAM) film excited by 285 nm UV light.

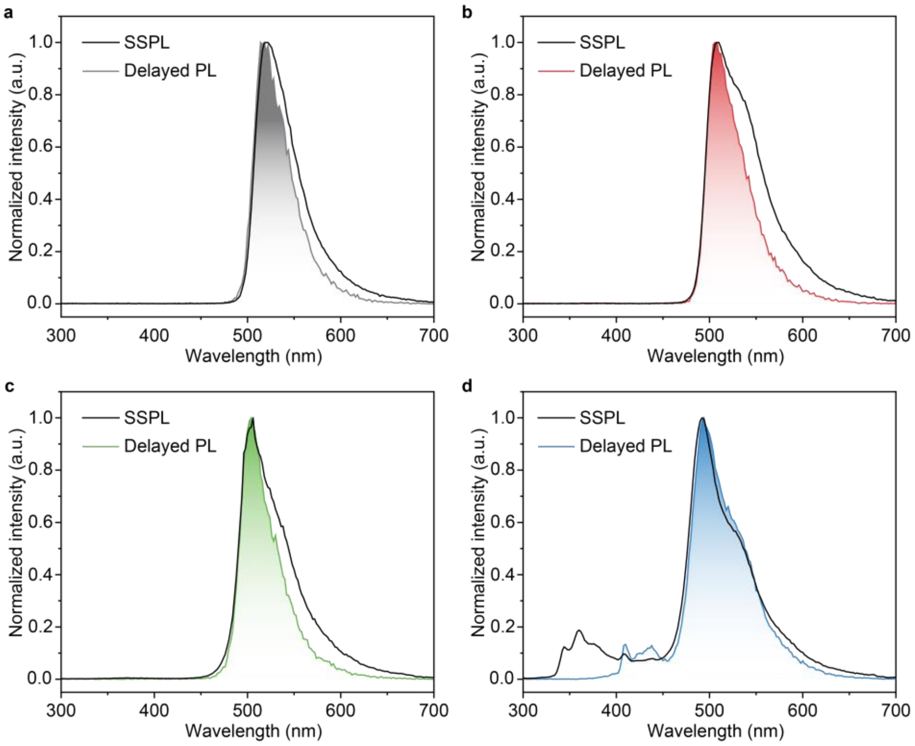

Supplementary Figure 22. SSPL and delayed PL (10 ms delay) spectra of (a) $PAMQA_1$, (b) $PAMQA_2$, (c) $PAMQA_3$ and (d) $PAMQA_4$ films excited by 285 nm UV light at 77 K.

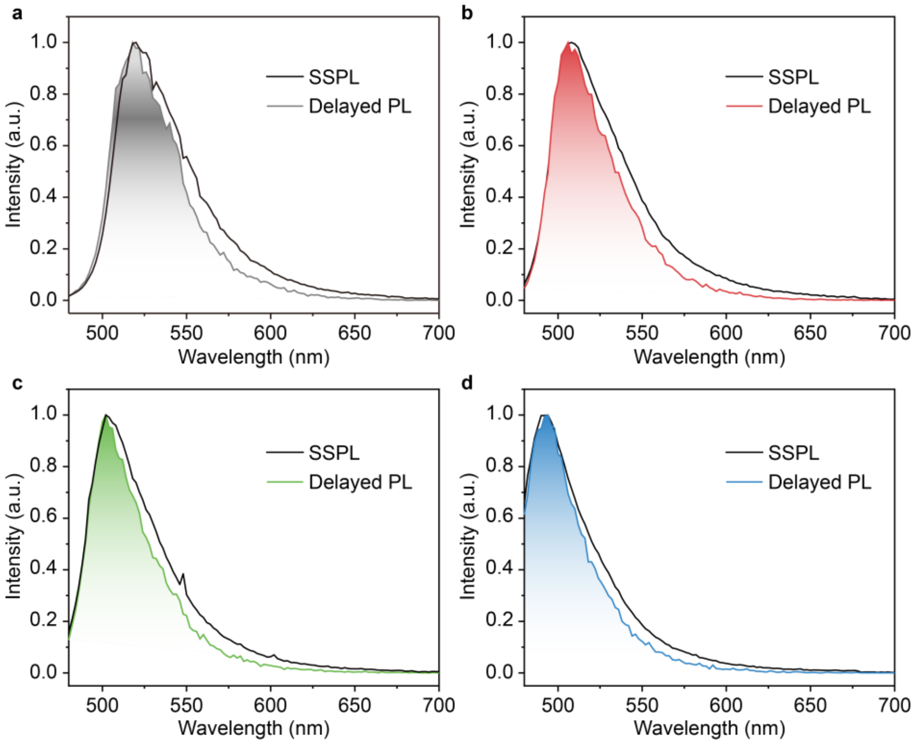

Supplementary Figure 23. SSPL and delayed PL (10 ms delay) spectra of (a) $PAMQA_1$, (b) $PAMQA_2$, (c) $PAMQA_3$ and (d) $PAMQA_4$ films excited by 466 nm light at 77 K.

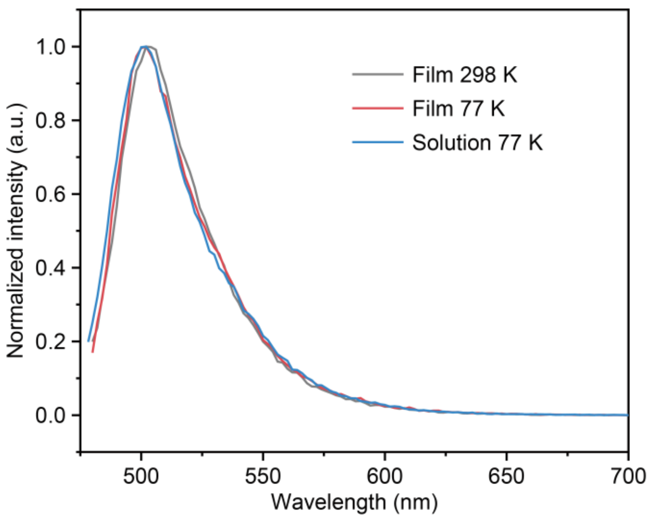

Supplementary Figure 24. Delayed PL (10 ms delay) spectra of $PAMQA_3$ film, solution (0.1 mg/mL) excited by 466 nm light at 298 K and 77 K.

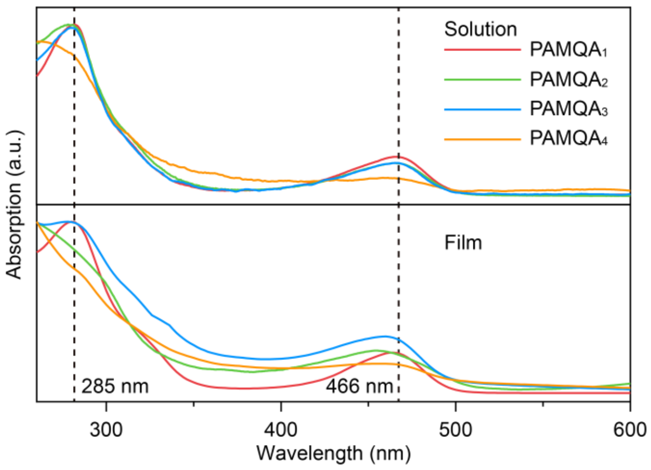

Supplementary Figure 25. UV-visible absorption spectra of $PAMQA_x$ (X=1, 2, 3 and 4) dilute aqueous solution (0.1 mg/mL) (top) and film (bottom) under ambient conditions.

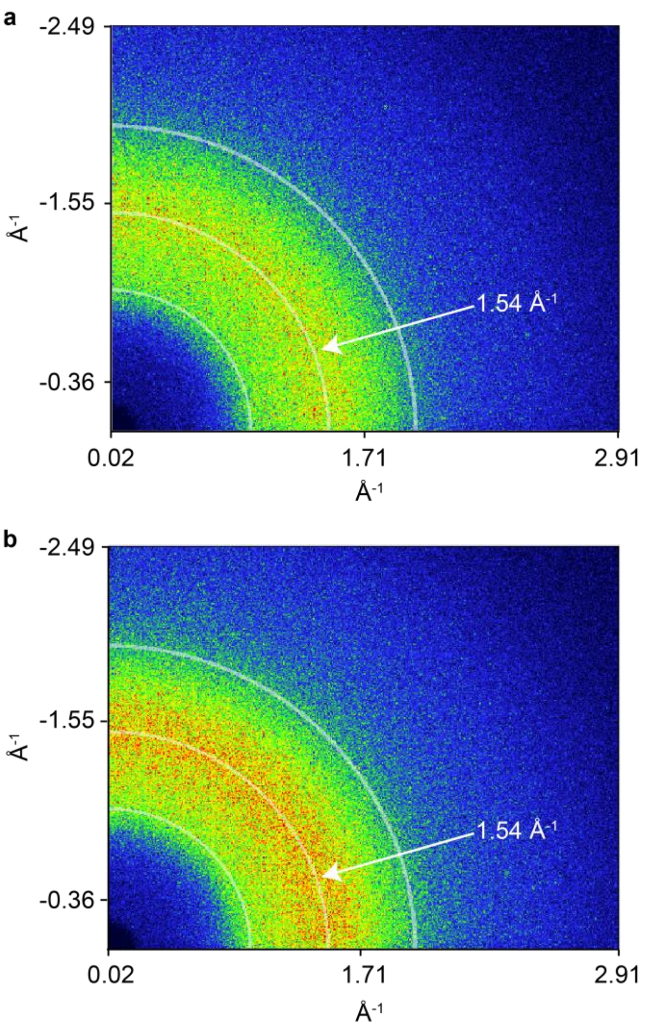

Supplementary Figure 26. 2D-WAXS patterns of (a) PAM and (b) $PAMQA_1$ films.

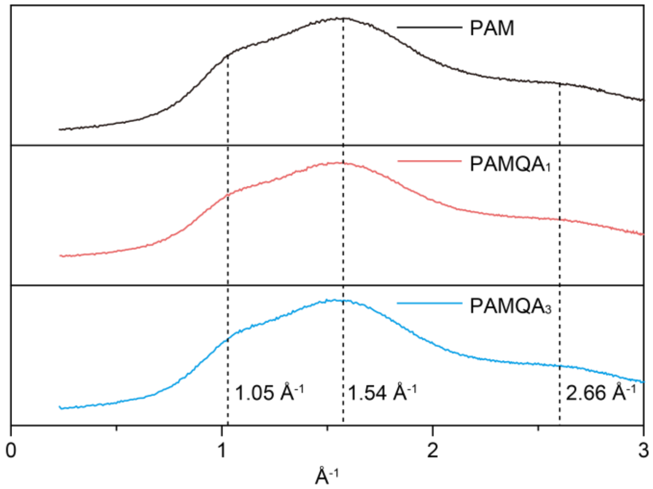

Supplementary Figure 27. WAXS spectra of PAM, $PAMQA_1$ and $PAMQA_3$ polymer films.

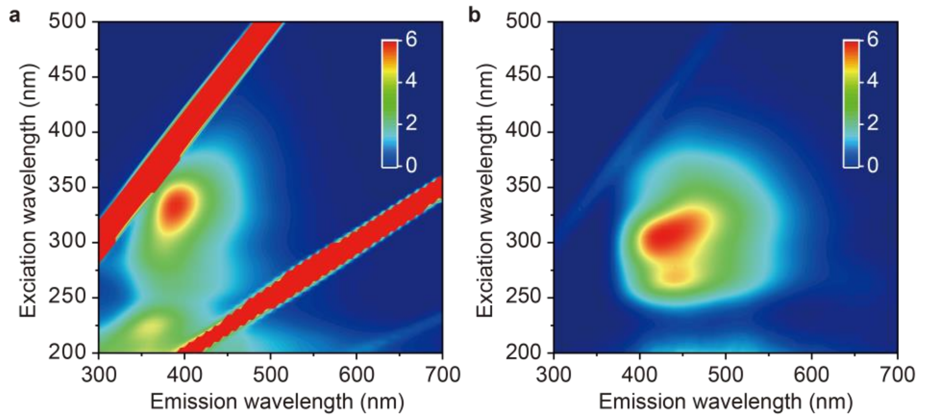

Supplementary Figure 28. (a) Excitation-SSPL and (b) excitation-delayed PL (25 ms delay) mappings of the PAM film under ambient conditions.

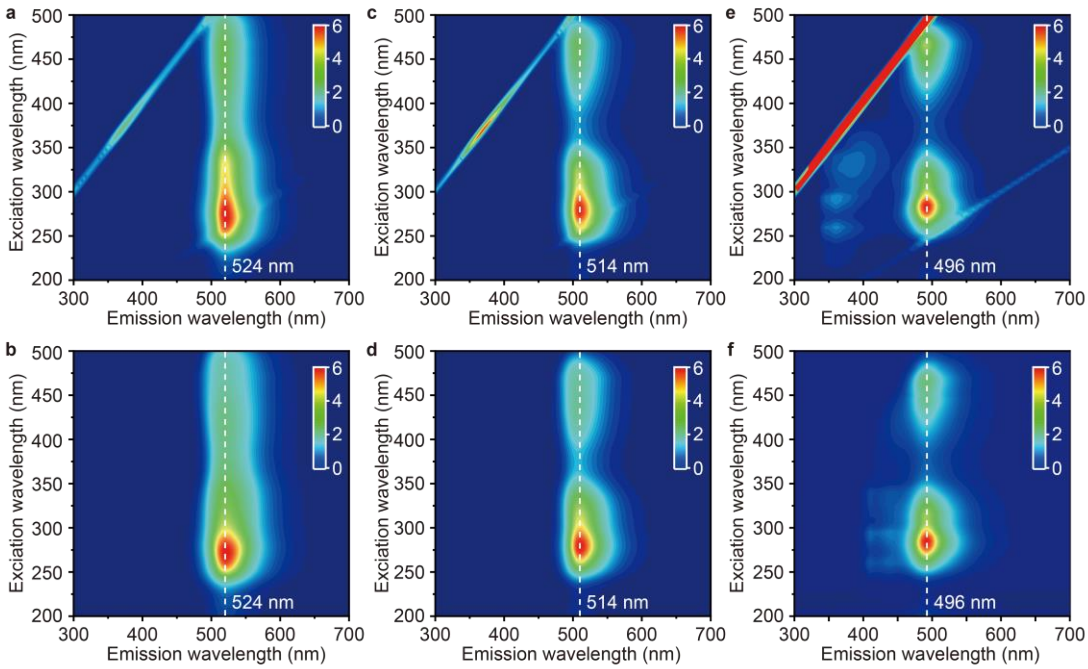

Supplementary Figure 29. (a, c, e) Excitation-SSPL and (b, d, f) excitation-delayed PL (25 ms delay) mappings of the (a, b) $PAMQA_1$, (c, d) $PAMQA_2$ and (e, f) $PAMQA_4$ films under ambient conditions.

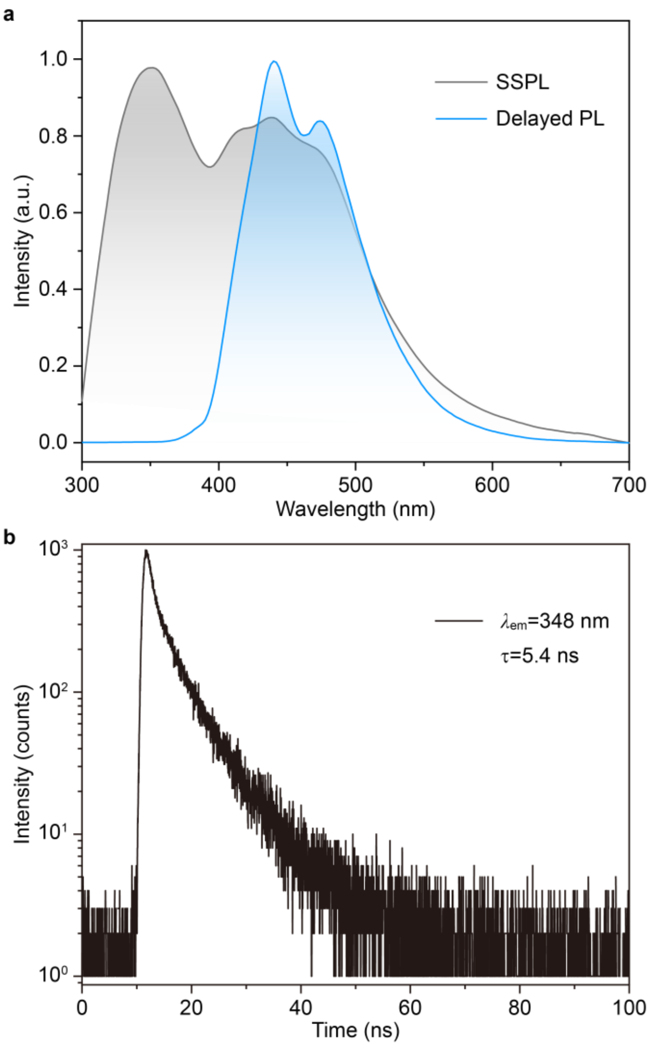

Supplementary Figure 30. (a) SSPL and delayed PL spectra of PAM film excited by 285 nm UV light under ambient conditions. (b) Fluorescence decay profiles of PAM film excited by 295 nm UV light under ambient conditions.

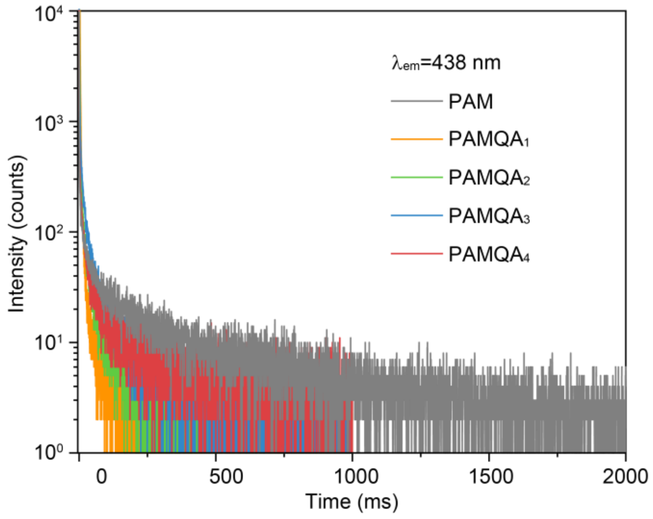

Supplementary Figure 31. Lifetime decay profiles of $PAMQA_x$ and PAM films upon 285 nm UV light excitation.

Supplementary Table 3. Phosphorescence amplitude lifetime and corresponding energy transfer efficiency of $PAMQA_x$.

Polymer	λ_P^H (nm)	$\tau_{amp}^{H, P}$ (ms)	λ_P^G (nm)	$\tau_{amp}^{G, P}$ (ms)	Φ_{FRET} (%)
PAM	438	170	--	--	--
$PAMQA_1$	438	9	524	15	94.7
$PAMQA_2$	438	11	514	21	93.5
$PAMQA_3$	438	20	504	30	88.2
$PAMQA_4$	438	23	496	34	86.5

H: host; G: guest; P: phosphorescence; Φ_{FRET}: energy transfer efficiency calculated from the equation, $\Phi_{RET} = 1 - \tau_{amp}^{H, P}/\tau_{amp}^{P}$, where $\tau_{amp}^{H, P}$ and τ_{amp}^{P} are the amplitude averaged lifetime of PAM and $PAMQA_x$ films.

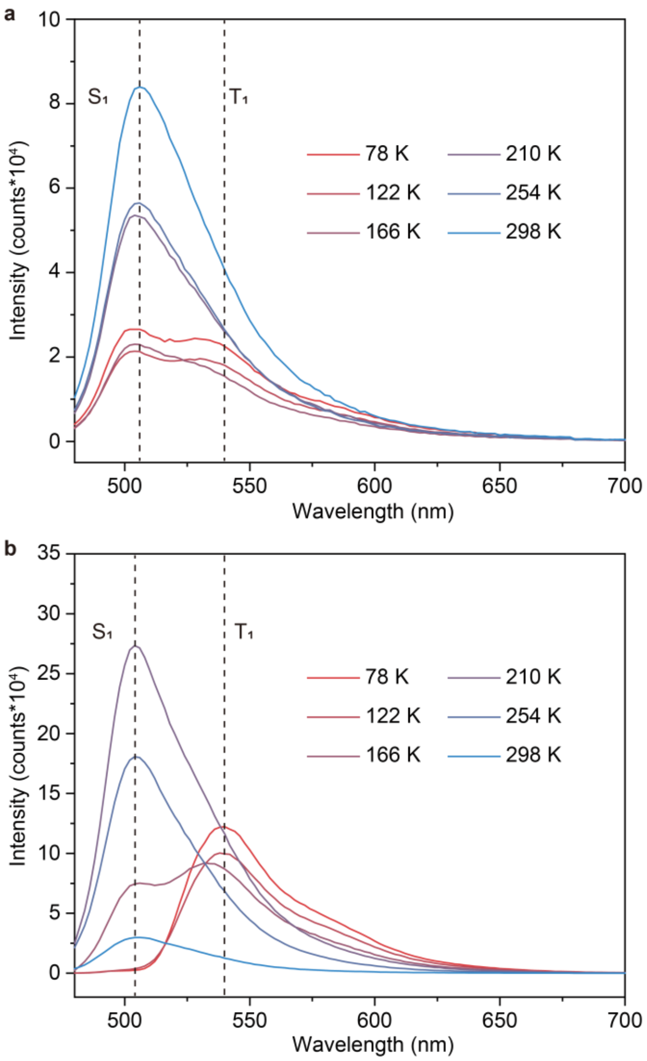

Supplementary Figure 32. Temperature-dependent (a) SSPL and (b) delayed PL (10 ms delay) spectra of $PAMQA_3$ film from 298 to 78 K excited by 466 nm visible light under argon condition.

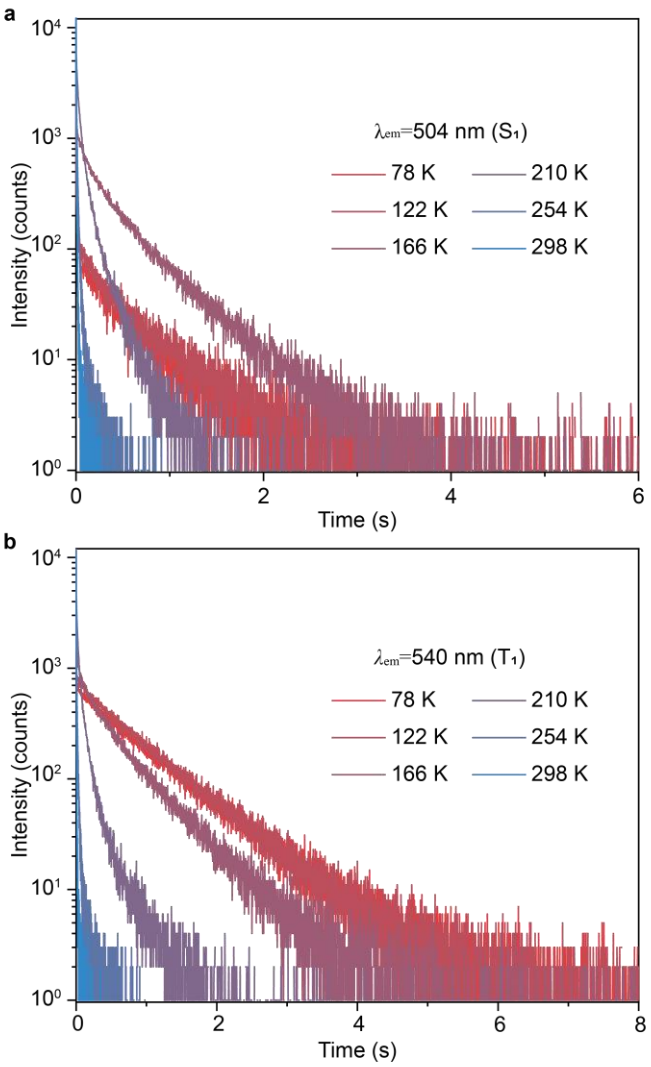

Supplementary Figure 33. Temperature-dependent lifetime decay profiles of emission bands at (a) 504 nm and (b) 540 nm of $PAMQA_3$ film excited by 285 nm UV light under argon condition.

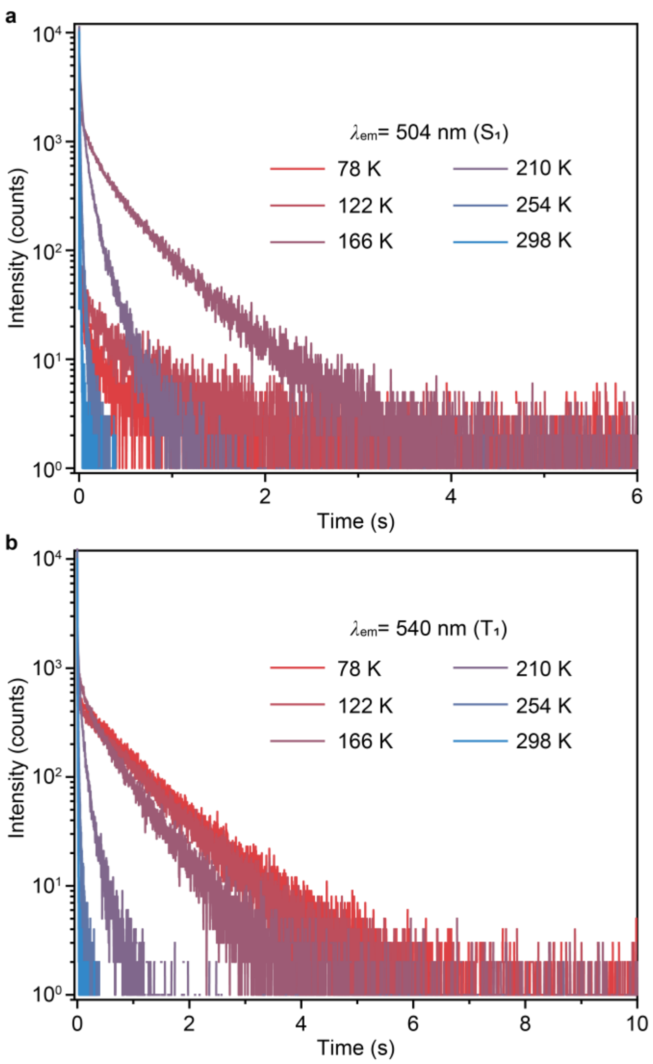

Supplementary Figure 34. Temperature-dependent lifetime decay profiles of emission bands at (a) 504 nm and (b) 540 nm of $PAMQA_3$ film excited by 466 nm light under argon condition.

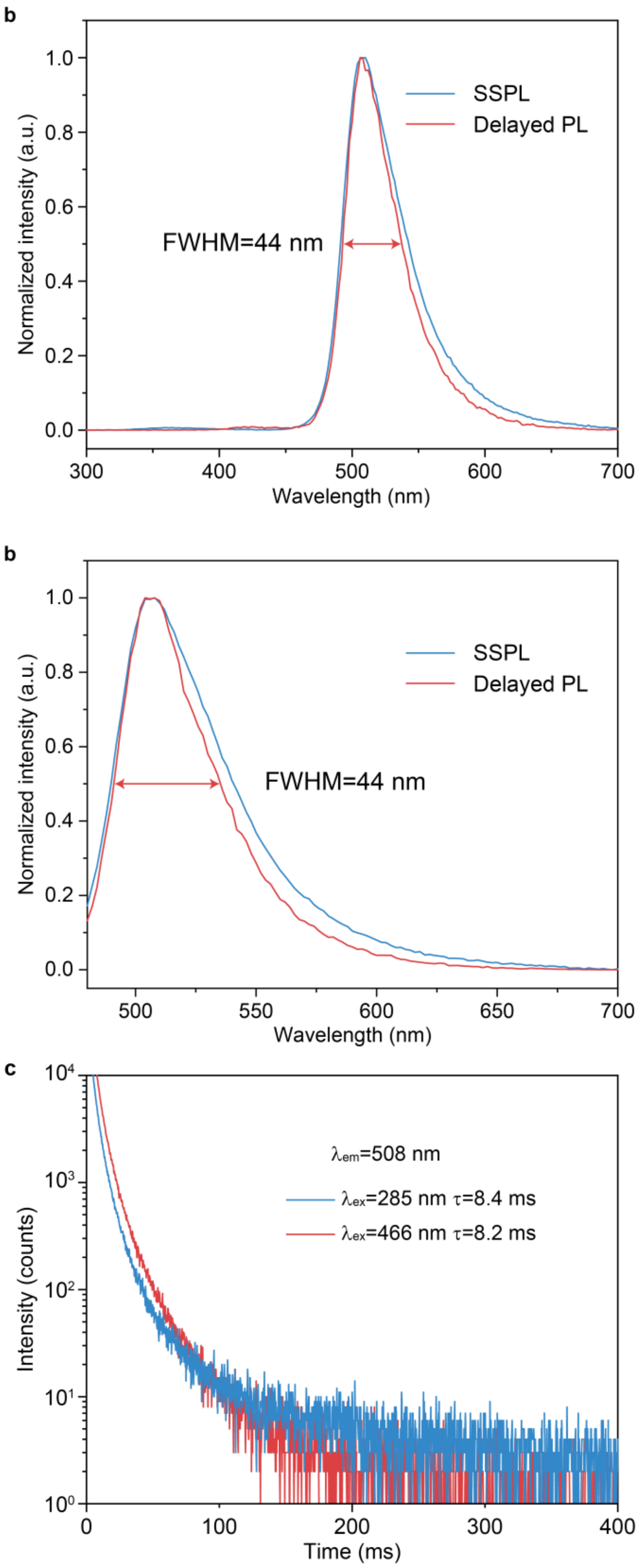

Supplementary Figure 35. SSPL and delayed PL spectra (10 ms delay) of PAAQA film upon (a) 285 nm UV light and (b) 466 nm visible light excitation. (c) Lifetime decay profiles of emission band at 508 nm of PAAQA film upon 285 nm UV light and 466 nm visible light excitation.

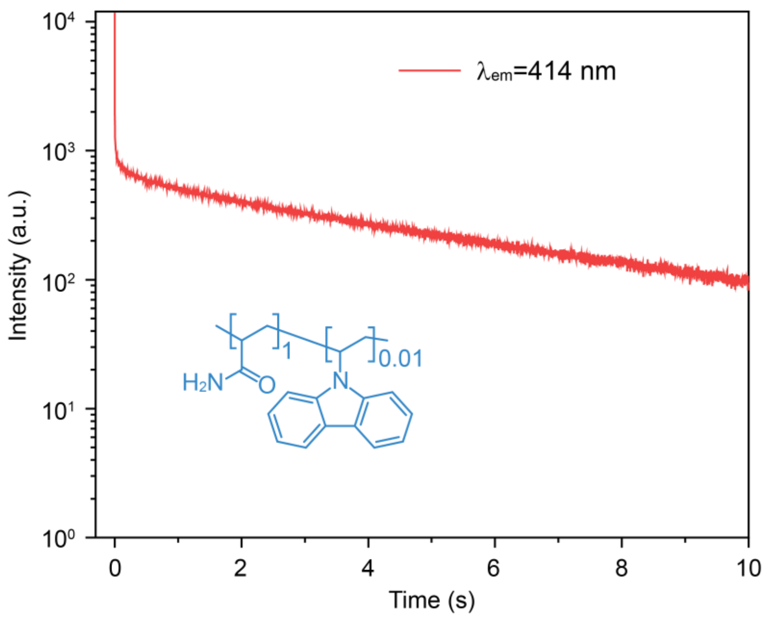

Supplementary Figure 36. Lifetime decay profile of the afterglow emission band at 414 nm of PAMCz film. Inset shows molecule structure of PAMCz.

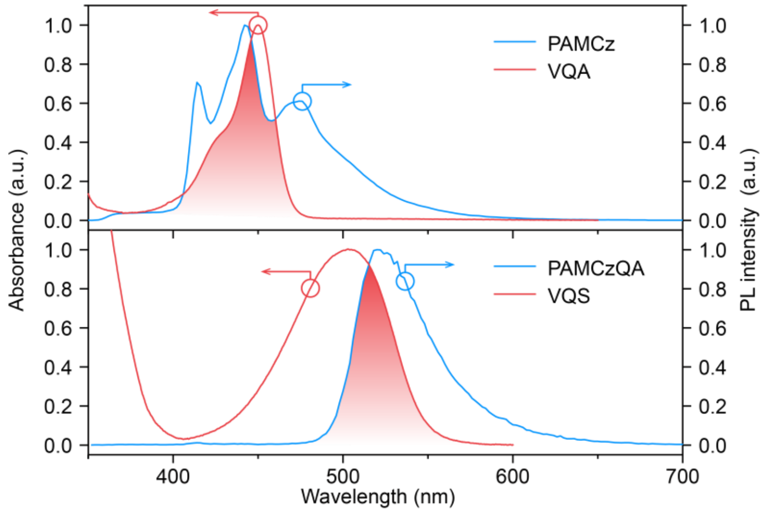

Supplementary Figure 37. Delayed PL spectra (10 ms delay) of PAMCz and PAMCzQA films as well as absorption spectra of VQA and VQS in toluene solution.

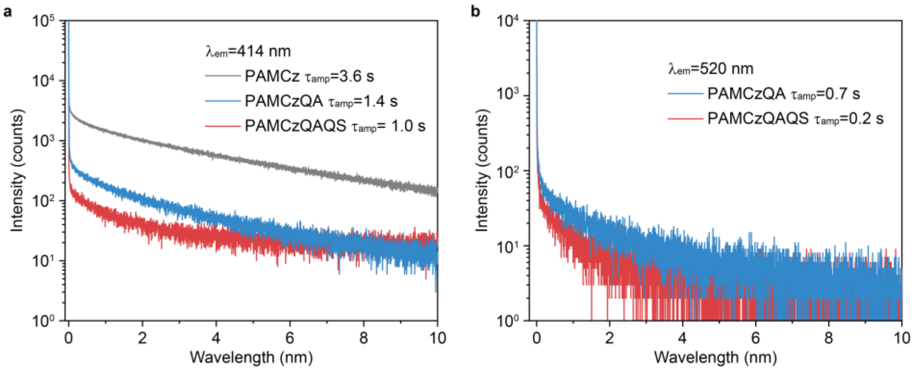

Supplementary Figure 38. Lifetime decay profiles of PAMCz, PAMCzQA and PAMCzQAQS at the emission bands of (a) 414 and (b) 520 nm upon 285 nm UV light excitation.

Supplementary Table 4. Phosphorescence amplitude lifetime and corresponding energy transfer efficiency of PAMCzQA and PAMCzQAQS.

Polymer	λ_P^{H} (nm)	$\tau_{amp}^{H, P}$ (s)	λ_P^{VQA} (nm)	$\tau_{amp}^{VQA,}$ (s)	$\Phi_{P\text{-}FRET}$ (%)
PAMCz	414	3.6	--	--	--
PAMCzQA	414	1.4	520	0.7	61.1
PAMCzQAQS	414	1.0	520	0.2	72.2

3. Hyperafterglow LED and displays

Fabrication and measurements of hyperafterglow LED devices: In a general procedure, $PAMQA_3$ was dissolved in a water solution (100 mg/mL) and then the $PAMQA_3$ solution was coated on a self-designed lampshade. Then the lampshade with $PAMQA_3$ film was assembled with a UV LED chip (285 nm) for fabricating the prototype hyperafterglow LED. The devices without encapsulation were measured immediately after fabrication under ambient atmosphere at room temperature. Steady-state electroluminescent (EL) spectra of the devices were measured by a PR655 spectra scan spectrometer and delayed EL (10 ms) spectra of the devices were measured using an Edinburgh FLS980 fluorescence spectrophotometer. The luminance-voltage and current-voltage characteristics were recorded using an optical power meter and a Keithley 2602 voltage current source.

Detailed procedure for the fabrication of hyperafterglow display panel: 5 g $PAMQA_3$ powders were dissolved in 15 mL deionized water, followed by the sonication for 30 mins under ambient conditions. Subsequently, the mixture was vigorously stirred at 60°C for 1 hour to obtain the transparent solution. Last, the well-mixed solution was poured into a clean teflon box, followed by natural evaporation of water. The large and uniform polymer film could be easily achieved for display applications.

The operation principle of circuitry-controlled LED array: Firstly, we assembled an UV LED array into the acrylic template (**Supplementary Figs. 40-41)**, then the UV LED array was connected and controlled by commercial programmable LED controller and transformer. Through programming controller, varied digit numbers and paths for display applications can be conveniently achieved by modulating the circuitry-controlled LED array. For example, by manipulating the column of the control table, the digital numbers 0-9 can be easily achieved, and then by programming the row of the control table, the drive duration and interval time for digital numbers 0-9 can be precisely modulated. When the No.1-No.6 and No.10-No.15 LEDs of the column are red (**Supplementary Figure. 42**), the digital number 0 is lighting and continuous lights for 6 seconds (time value 1), then the LED array extinguishes for 6 seconds, and the next digit number will light for another 6 seconds (time value 1).

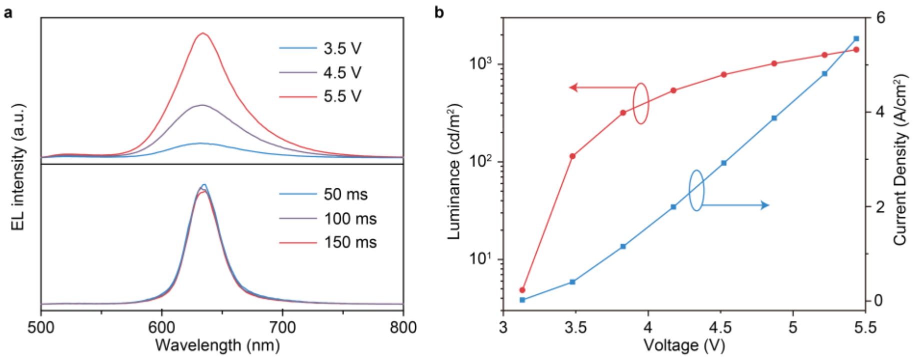

Supplementary Figure 39. (a) SSEL (top panel) and delayed EL (bottom panel) spectra of red hyperafterglow LED at varied driving voltages (top panel) and delayed times (bottom panel). (b) Current density-voltage-luminescence curves of red hyperafterglow LED.

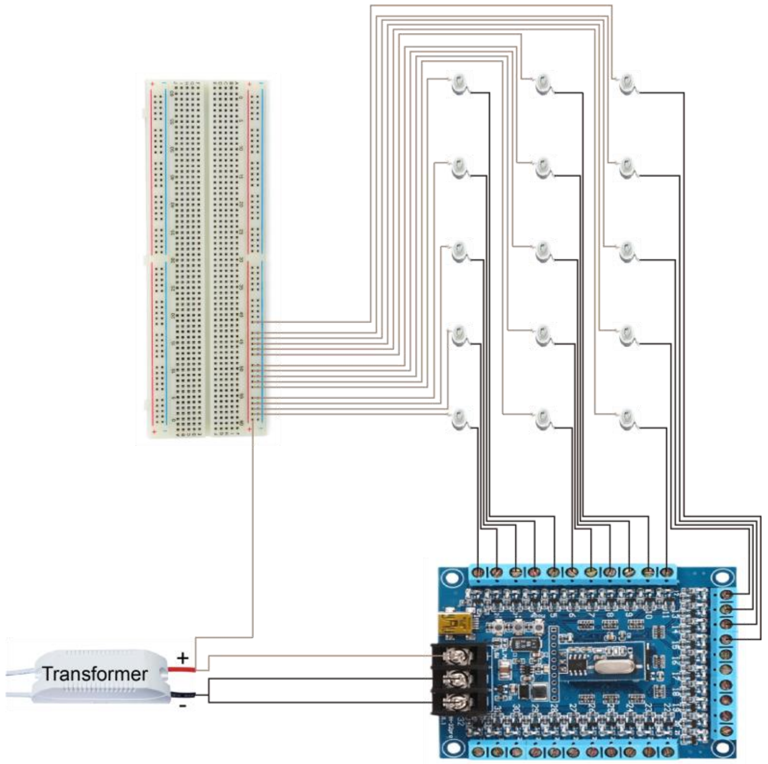

Supplementary Figure 40. Circuit diagram of the afterglow display applications for digital pattern.

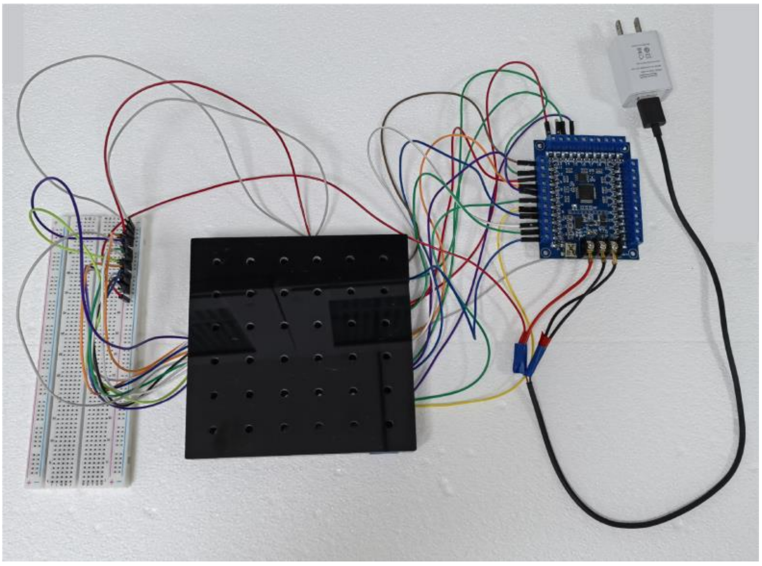

Supplementary Figure 41. Photograph of the assembled DC-driven digital display application.

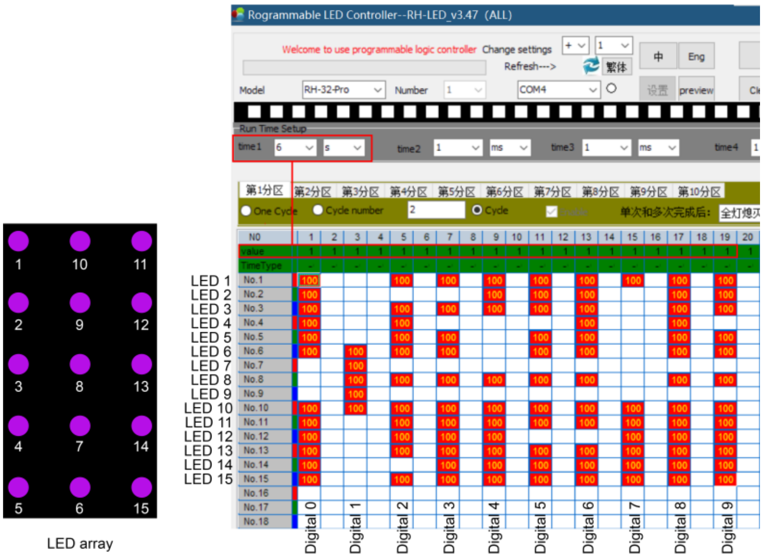

Supplementary Figure 42. Program of the control of drive duration and interval time of display for digital number 0-9.

Supplementary References

1. Qiu, X. et al. Narrowband emission from organic fluorescent emitters with dominant low‐frequency vibronic coupling. *Adv. Opt. Mater.* **9**, 2001845 (2021).
2. Zou, S. N. et al. Fully bridged triphenylamine derivatives as color-tunable thermally activated delayed fluorescence emitters. *Org. Lett.* **23**, 958-962 (2021).
3. Peng, H. et al. On-demand modulating afterglow color of water-soluble polymers through phosphorescence FRET for multicolor security printing. *Sci. Adv.* **8**, eabk2925 (2022).
4. Jin, J. B. et al. Thermally activated triplet exciton release for highly efficient tri-mode organic afterglow. *Nat. Commun.* **11**, 842 (2020).
5. Hamzehpoor, E. & Perepichka, D. F. Crystal engineering of room temperature phosphorescence in organic solids. *Angew. Chem. Int. Edit.* **59**, 9977-9981 (2020).
6. Zhao, W. J. et al. Boosting the efficiency of organic persistent room-temperature phosphorescence by intramolecular triplet-triplet energy transfer. *Nat. Commun.* **10**, 1595 (2019).
7. Gu, L. et al. Colour-tunable ultra-long organic phosphorescence of a single-component molecular crystal. *Nat. Photon*. **13**, 406-411 (2019).
8. Yang, Z. et al. Boosting the quantum efficiency of ultralong organic phosphorescence up to 52 % via intramolecular halogen bonding. *Angew. Chem. Int. Edit.* **59**, 17451-17455 (2020).
9. Ma, X. K. et al. Supramolecular pins with ultralong efficient phosphorescence. *Adv. Mater.* **33**, 2007476 (2021).
10. Ma, L. W., Sun, S. Y., Ding, B. B., Ma, X. & Tian, H. Highly efficient room-temperature phosphorescence based on single-benzene structure molecules and photoactivated luminescence with afterglow. *Adv. Funct. Mater.* **31**, 2010659 (2021).
11. Ye, W. P. et al. Confining isolated chromophores for highly efficient blue phosphorescence. *Nat. Mater.* **20**, 1539 (2021).
12. Zheng, X. et al. Nearly unity quantum yield persistent room-temperature phosphorescence from heavy atom-free rigid inorganic/organic hybrid frameworks. *Angew. Chem. Int. Edit.* **61**, e202207104 (2022).
13. Wang, X. P. et al. TADF-type organic afterglow. *Angew. Chem. Int. Edit.* **60**, 17138-17147 (2021).
14. Xu, S. et al. Design of highly efficient deep-blue organic afterglow through guest sensitization and matrices rigidification. *Nat. Commun.* **11**, 4802 (2020).
15. Wang, D. H. et al. Boric acid-activated room-temperature phosphorescence and thermally activated delayed fluorescence for efficient solid-state photoluminescence materials. *Adv. Opt. Mater.***10**, 2200629 (2022).
16. Bolton, O., Lee, K., Kim, H., Lin, K. Y. & Kim, J. Activating efficient phosphorescence from purely organic materials by crystal design. *Nat. Chem.* **3**, 205-210 (2011).
17. Gu, L. et al. Color-tunable ultralong organic room temperature phosphorescence from a multicomponent copolymer. *Nat. Commun.* **11**, 944 (2020).
18. Bhattacharjee, I. & Hirata, S. Highly efficient persistent room-temperature phosphorescence from heavy atom-free molecules triggered by hidden long phosphorescent antenna. *Adv. Mater.* **32**, 2001348 (2020).
