# Supplementary material for: Multicolor hyperafterglow from isolated fluorescence chromophores
Source: Nat Commun. 2023 Jan 30;14:475. doi: 10.1038/s41467-023-36105-y (PMC9884663; doi:10.1038/s41467-023-36105-y)
Supplement: Supplementary file 3 — Description of Additional Supplementary Files [file 41467_2023_36105_MOESM3_ESM.pdf]

Description of Additional Supplementary Files

File Name: Supplementary Movie 1
Description: Path display from A to B

File Name: Supplementary Movie 2
Description: NJUPT characters display
